# Inorganic Chemistry of the Tripodal Picolinate Ligand **Tpaa** with Gallium(III) and Radiolabeling with Gallium-68

**DOI:** 10.1021/acs.inorgchem.3c02459

**Published:** 2023-10-04

**Authors:** Thomas W. Price, Laurène Wagner, Veronika Rosecker, Jana Havlíčková, Timothy J. Prior, Vojtěch Kubíček, Petr Hermann, Graeme J. Stasiuk

**Affiliations:** † Department of Imaging Chemistry and Biology, School of Biomedical Engineering and Imaging Sciences, King’s College London, London SE1 7EH, United Kingdom; ‡ Department of Inorganic Chemistry, Faculty of Science, Charles University, Hlavova 8, 128 40 Prague 2, Czech Republic; § Chemistry, School of Natural Sciences, University of Hull, Cottingham Road, Hull HU6 7RX, United Kingdom

## Abstract

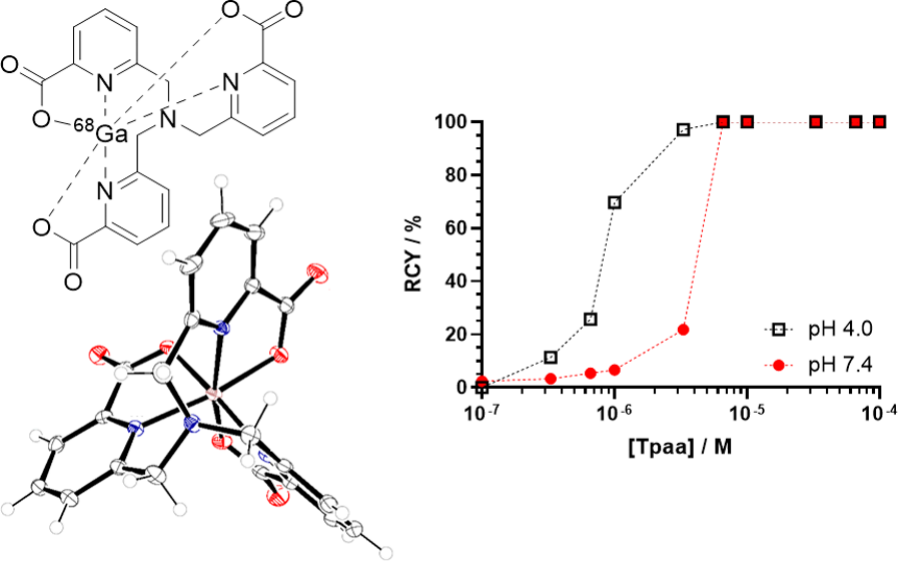

We report here the improved synthesis of the tripodal picolinate chelator **Tpaa**, with an overall yield of 41% over five steps, in comparison to the previously reported 6% yield. **Tpaa** was investigated for its coordination chemistry with Ga(III) and radiolabeling properties with gallium-68 (^68^Ga). The obtained crystal structure for [Ga(**Tpaa**)] shows that the three picolinate arms coordinate to the Ga(III) ion, fully occupying the octahedral coordination geometry. This is supported by ^1^H NMR which shows that the three arms are symmetrical when coordinated to Ga(III). Assessment of the thermodynamic stability through potentiometry gives log *K*_Ga-**Tpaa**_ = 21.32, with a single species being produced across the range of pH 3.5–7.5. **Tpaa** achieved >99% radiochemical conversion with ^68^Ga under mild conditions ([**Tpaa**] = 6.6 μM, pH 7.4, 37 °C) with a molar activity of 3.1 GBq μmol^–1^. The resulting complex, [^68^Ga][Ga(**Tpaa**)], showed improved stability over the previously reported [^68^Ga][Ga(**Dpaa**)(H_2_O)] in a serum challenge, with 32% of [^68^Ga][Ga(**Tpaa**)] remaining intact after 30 min of incubation with fetal bovine serum.

## Abstract 2

**Tpaa** fully occupies the octahedral geometry of Ga(III), allowing for improved stability and radiolabeling characteristics in comparison to the previously reported **Dpaa**. Synthesis of **Tpaa** was achieved in a 41% yield over 5 steps. The three picolinate arms of **Tpaa** coordinate to Ga(III) in a symmetrical manner, yielding a complex with log *K*_Ga-**Tpaa**_ = 21.32. **Tpaa** can be radiolabeled under mild conditions (pH 7.4, [*L*] = 6.6 μM, *T* = 37 °C).

## Introduction

Generator-produced gallium-68 (^68^Ga) is an exciting isotope for positron emission tomography (PET),^1,2^ a highly sensitive imaging modality. The benchtop production of ^68^Ga using a generator has the potential for application in the kit-type production of radiotracers for medical application, as has been seen for the widely popular technetium-99m.^3^ This would enable the realization of PET in hospitals and other institutes without direct access to the extensive infrastructure required for other radioisotopes, such as fluorine-18.

**Figure 1:**
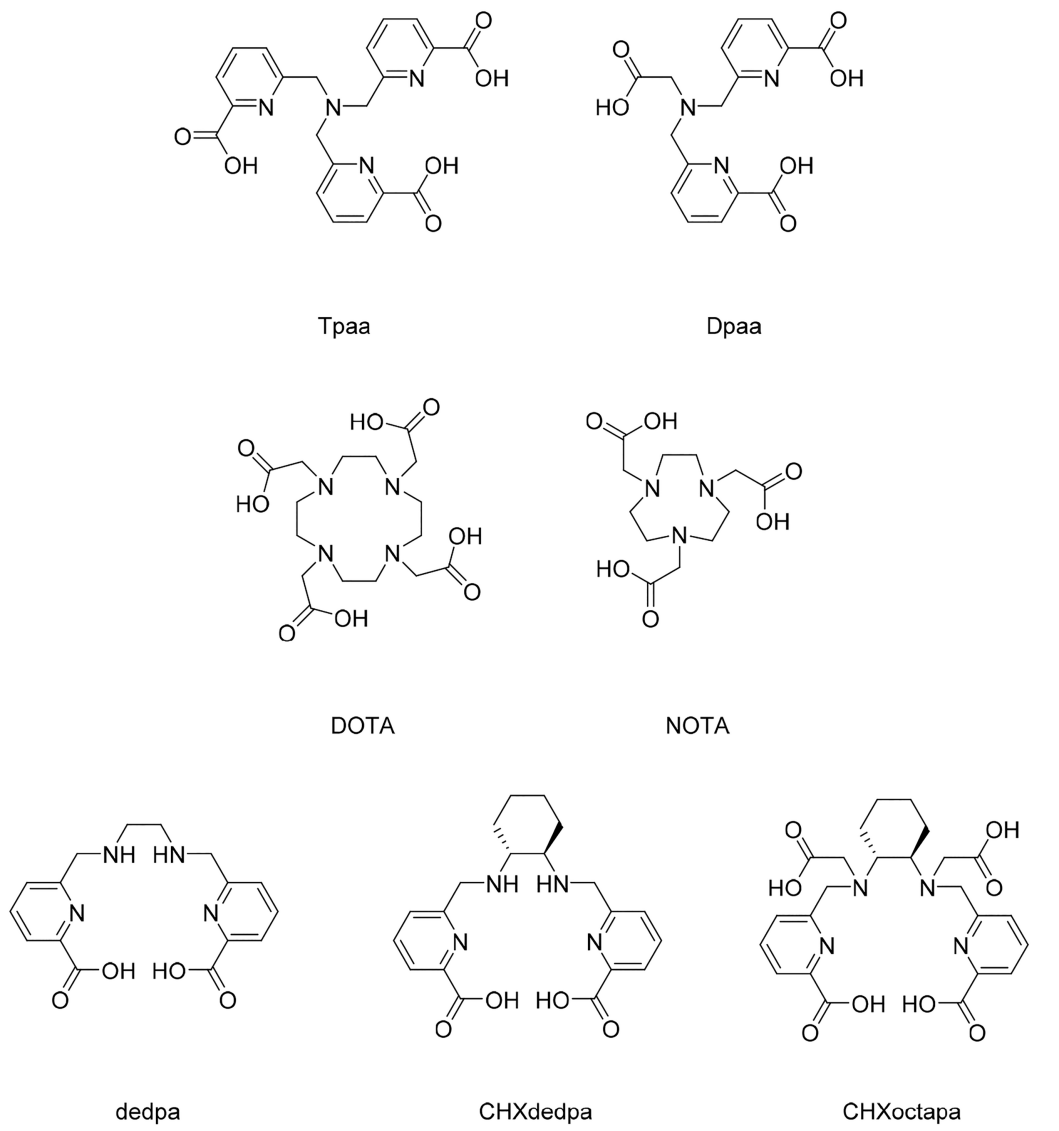
Structures of chelators discussed in this manuscript.

The most widely used chelate for ^68^Ga is **DOTA** (1,4,7,10-tetraazacyclododecane-1,4,7,10-tetraacetic acid, Figure 1).^4,5^ Conjugates of **DOTA** have been applied to ^68^Ga PET of a variety of targets,^6,7^ with particular success seen for somatostatin targeting probes such as **DOTATATE**.^8^ However, radiolabeling conditions for **DOTA** with ^68^Ga are harsh, requiring acidic conditions (pH 4.0) and high temperatures (80 °C) for efficient radiolabeling, due to the poor size match of the cavity of the **DOTA** ligand for Ga(III) coordination.^4,9,10^ Recent developments in ^68^Ga chelate design have improved the metal–ligand match, and this has resulted in milder radiolabeling conditions being used for efficient radiolabeling.^11−15^ The choice of chelator has been reported to have an impact upon the localization and clearance of the radiotracer;^16−21^ therefore, having a library of efficient chelators will aid in the rapid development of novel radiotracers with optimized uptake in target tissues.

To contribute to a library of chelators that can be labeled with ^68^Ga under mild conditions, we have recently reported the complexation of ^68^Ga by the tripodal picolinate chelate **Dpaa** (6,6′-{[(carboxymethyl)azanediyl]bis(methylene)}dipicolinic acid, Figure 1) and bifunctional derivatives.^22,23^ This system was also reported by the Orvig group.^24^ While **Dpaa** could be radiolabeled at neutral pH, the serum stability of the resulting complex was unsatisfactory for *in vivo* imaging, with [^67/68^Ga][Ga(**Dpaa**)(H_2_O)] being reported as being only 58% intact after 2 h in 50% human serum^24^ and being completely decomplexed within 30 min in 90% fetal bovine serum.^22^

Other chelators for ^67/68^Ga based on picolinate units have also been reported.^25−27^ The linear chelator **dedpa** (6,6′-{[ethane-1,2-diylbis(azanediyl)]bis(methylene)}dipicolinic acid, Figure 1 and Table 1) was shown to label at very low ligand concentrations under acidic conditions,^25^ with the resulting complex remaining 77.8% intact after 2 h in 50% human serum.^26^ A more rigid version of this chelator, **CHXdedpa** (6,6′-{[((1*R*,2*R*)-cyclohexane-1,2-diyl)bis(azanediyl)]bis(methylene)}dipicolinic acid, Figure 1), required slightly higher ligand concentrations for high radiochemical conversion (RCC) but yielded a complex that was more resistant to decomplexation, with 90.5% [^67^Ga][Ga(**CHXdedpa**)] remaining intact after 2 h in 50% human serum.^26^ Increasing the number of coordinating atoms to 8, by adding two acetate arms to form **CHXoctapa** (6,6′-{[((1*R*,2*R*)-cyclohexane-1,2-diyl)bis({carboxymethyl}azanediyl)]bis(methylene)}dipicolinic acid, Figure 1), reduced the stability of the resulting ^67^Ga complex to 74.7% after 2 h in 50% human serum.^26^

**Table 1:** Radiolabeling Parameters for Chelators Discussed with ^68^Ga

	radiolabeling conditions					
chelator	[*L*]/M	pH	*T*/°C	*t*/min	RCC/%	stability to serum/%
**Dpaa**	10^–4^	7.4	37	15	95^22^	0,^a^^,^^22^, 58^b^^,^^24^
**DOTA**	10^–5^	4–5	80	5	95.2^28^	80.0^b^^,^^24^
**NOTA**	10^–6^	4–5	25	5	98.6^28^	98.0^b^^,^^24^
**dedpa**	10^–7^	4.5	25	10	>99^25^	77.8^b^^,^^26^
**CHXdedpa**	10^–5^	4.0	25	10	>99^26^	90.5^b^^,^^26^
**CHXoctapa**	10^–5^	4.0	25	10	>99^26^	74.7^b^^,^^26^

a Conditions: 90% serum, 30 min, 37 °C.

b Conditions: 50% serum, 2 h, 37 °C.

These results encouraged us to develop chelators based upon the **Dpaa** system to improve the stability of the ^68^Ga complex upon exposure to serum. In the crystal structure of [Ga(**Dpaa**)(H_2_O)], the central amine of the **Dpaa** ligand was shown not to coordinate to the Ga(III) center,^22,24^ resulting in one of the coordination sites of the metal being occupied by water, and further evidence of this was seen through potentiometry.^22,24^ While examples of stable ^68^Ga systems featuring coordinated water or hydroxide molecules have been reported,^29,30^ in the case of the Ga-**Dpaa** system this is a potential cause of the low stability of the complex in biological media. With a view to developing the tripodal picolinate family of ligands further, we have investigated a picolinate chelate with an increased number of coordinating atoms, **Tpaa** (6,6′,6″-(nitrilotris(methylene))tripicolinic acid, Figure 1). These additional coordinating atoms should compensate for the noncoordinating central amine while retaining the favorable radiolabeling properties of the picolinate functionalities.

Complexes of **Tpaa** have been previously reported with a variety of other metals; this ligand has been applied to the chelation of Pb^2+^,^31^ Mn^2+^,^32,33^ Ca^2+^,^31−35^ and a series of Ln^3+^ ions (Ln = La, Pr, Nd, Eu, Gd, Tb, Ho, Tm, Yb, Lu),^34,35^ resulting in complexes with coordination numbers from 6 to 10, with the metal ion being coordinated by 5–7 ligand atoms. In many instances, additional molecules, such as water, are involved in the metal coordination.^31−35^

We herein report and characterize the novel Ga(III) complex with **Tpaa**. The **Tpaa** ligand should form a [Ga(**Tpaa**)] complex where the central Ga(III) ion is bound solely through the picolinate pendant arms and the amine group serves as a scaffold arranging all the donor atoms to form a coordination polyhedron close to the ideal octahedral geometry. The incorporation of an additional picolinate arm should increase the stability of the complex in comparison with the previously reported [Ga(**Dpaa**)(H_2_O)] and improve its potential to be used as a ^68^Ga ligand.

## Results and Discussion

### Ligand and Complex Synthesis

**Scheme 1:**
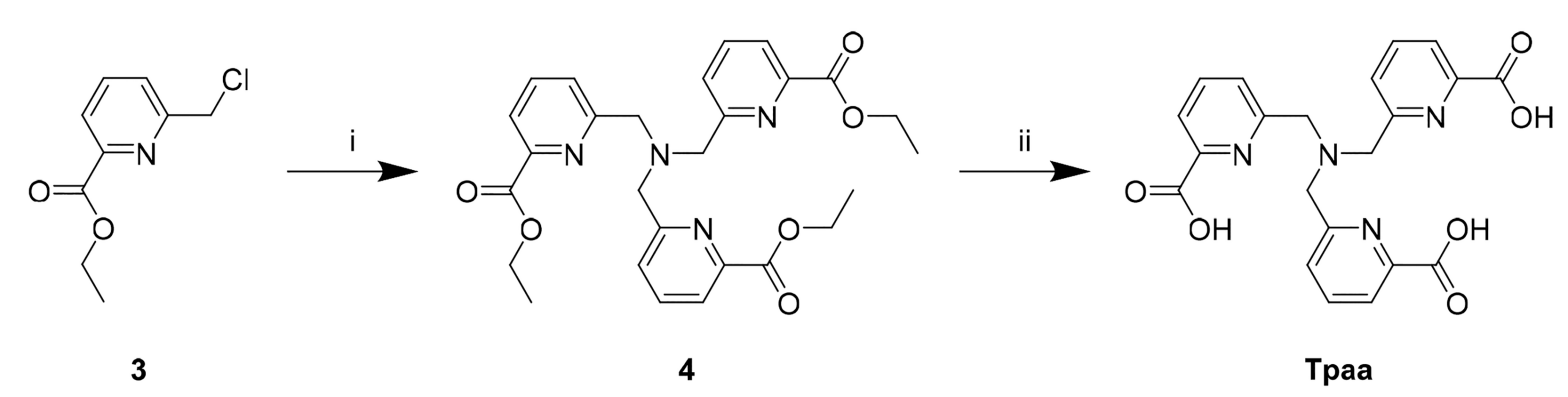
Synthesis of **Tpaa** Conditions: (i) NH_3_, MeCN, KI, RT-60 °C, 2 d; (ii) 6 M HCl, reflux, 16 h.

**Tpaa** was synthesized in two steps from a previously described precursor, ethyl 6-(chloromethyl)picolinate (**3**, Scheme 1; for a modified synthesis, see the Supporting Information);^36^ reaction with ammonia in acetonitrile followed by deprotection under acidic conditions yielded **Tpaa** in a 71% yield over two steps. This results in an overall yield of 41% from commercially available 2,6-pyridinedicarboxylic acid in five steps,^36,37^ in contrast to an overall yield of 6% over nine steps starting from 2,6-lutidine reported previously.^34^

Complexation of Ga(III) by this chelate was achieved at pH 4.5 overnight, with the formation of [Ga(**Tpaa**)] confirmed by high-resolution mass spectrometry (*m*/*z* = 489.0321, [M + H]^+^, 489.0320 expected for ^69^GaC_21_H_16_N_4_O_6_; Figure S16). The ^1^H NMR spectrum of [Ga(**Tpaa**)] displayed characteristic geminal coupling of the methylene group between the central amine and the picolinate arms (^2^*J*_HH_ = 17.4 Hz; Figure S12). The picolinate arms were shown to be symmetrical, suggesting complete coordination of the Ga(III) center by the picolinates.

### Crystal Structure

**Figure 2:**
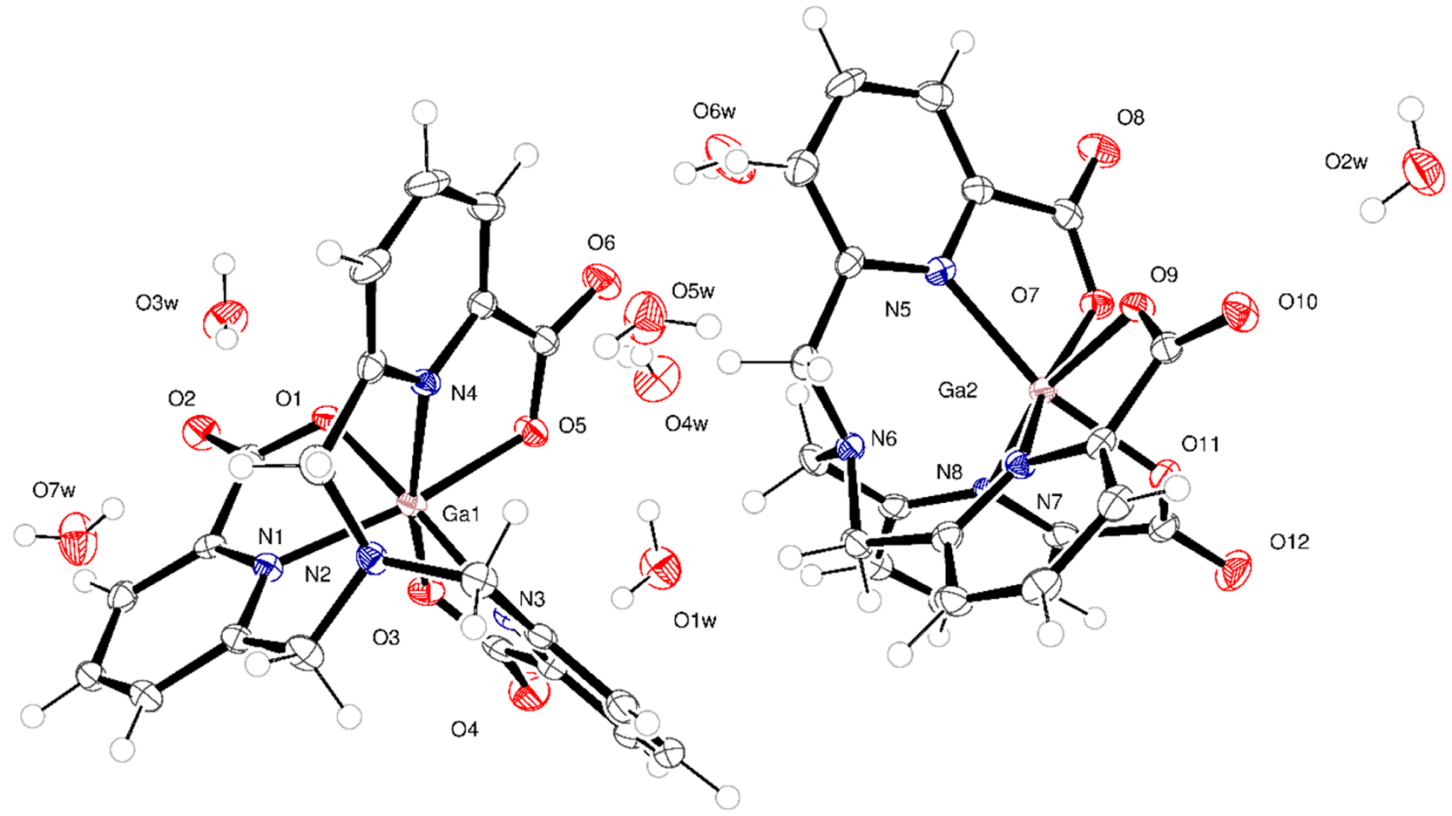
ORTEP representation of the molecular structure of [Ga(**Tpaa**)]_2_(H_2_O)_7_ with atoms drawn as 30% probability ellipsoids. Selected atoms are labeled. Full details are available in the Supporting Information.^a^

A crystal of suitable quality for X-ray diffraction analysis was obtained by the slow evaporation of an acidic solution of the complex.^a^ The structure crystallizes in the noncentric space group *P*2_1_. The asymmetric unit contained two symmetry-unique [Ga(**Tpaa**)] complexes and 7 water molecules (Figure 2). In both unique complexes, the Ga(III) center is coordinated by three chelating picolinate arms of the ligand; the overall coordination geometry is that of a distorted octahedron. The central amine of the ligand is not involved in complexation in this structure (Ga1···N2 distance 2.641(3) Å, Ga2···N6 distance 2.670(3) Å; Figure 2). This is in agreement with the previously reported structures for [Ga(**Dpaa**)(H_2_O)] in which the central amine is not involved in coordination of the Ga(III) ion (Ga–N distance 2.4880(11) Å).^22,24^

The Ga–N bond length of the coordinating picolinate arms is decreased (mean Ga–N bond length 2.08 Å) when compared to the bond length reported for [Ga(**Dpaa**)(H_2_O)] (mean Ga–N bond length 2.22 Å),^22^ suggesting an improved coordination; the Ga–O distance does not significantly change (mean Ga–O 2.01 vs 2.03 Å). This suggests that overall there is an improved interaction between the picolinate arms and the Ga(III) center. Furthermore, the mean picolinate bite angle is increased (78.66° compared to 74.46°)^22^—this is closer to the ideal 90° for an octahedral geometry, showing reduced strain in the [Ga(**Tpaa**)] complex than in [Ga(**Dpaa**)(H_2_O)]. There is no evidence for a coordinated water molecule, in contrast to the reported crystal structures for [Ga(**Dpaa**)(H_2_O)]. Further details of the crystal structure are contained in the Supporting Information.

### Thermodynamic Stability

Protonation constants of ligand **Tpaa** (Table 2, Table S2, and Figure S20A) were determined by potentiometry. Four protonation constants were found in the studied pH range. The first protonation is likely localized on the amine nitrogen atom; the remaining three protonations occur on pyridine or carboxylate groups and they cover the whole acidic region. The determined protonation constants are in a good agreement with the previously published data.^35^ Overall, the ligand basicity is rather low, which might be beneficial for complexation of metal radioisotopes in acidic solutions.

**Table 2:** Comparison of Stepwise Protonation and Stability Constants of the Discussed Ligands

constant	**Tpaa**^a^	**Tpaa**^b^	**Dpaa**^c^	**DOTA**^d^	**NOTA**^e^
log *K*_1_	6.95	6.78	7.38	11.9	13.17
log *K*_2_	4.20	4.11	3.73	9.72	5.74
log *K*_3_	3.36	3.3	2.82	4.60	3.22
log *K*_4_	2.05	2.5		4.13	1.96
log *K*_5_				2.36	0.7
log *K*_GaL_	21.32		18.53	26.05^37^	29.63^39^
pGa	9.19^f^^,^^g^			7.23^f^^,^^g^	11.82^f^^,^^g^
log *K*_CuL_	16.39		10.85		23.33
pCu	9.63^g^				10.28^g^

a This work, 25 °C, *I* = 0.1 M NMe_4_Cl.

b Reference (34), 0, *I* = 0.1 M KCl.

c Reference (21), *I* = 0.1 M NMe_4_Cl, *T* = 25 °C.

d Reference (37), *I* = 0.1 M NMe_4_Cl, *T* = 25 °C.

e References (38−40), *I* = 0.1 M NMe_4_Cl, *T* = 25 °C.

f Expressed as a negative logarithm of the [Ga(OH)_4_]^−^ concentration.

g *c*_M_ = 0.1 mM, *c*_L_ = 1 mM, pH 7.4, 25 °C.

**Figure 3:**
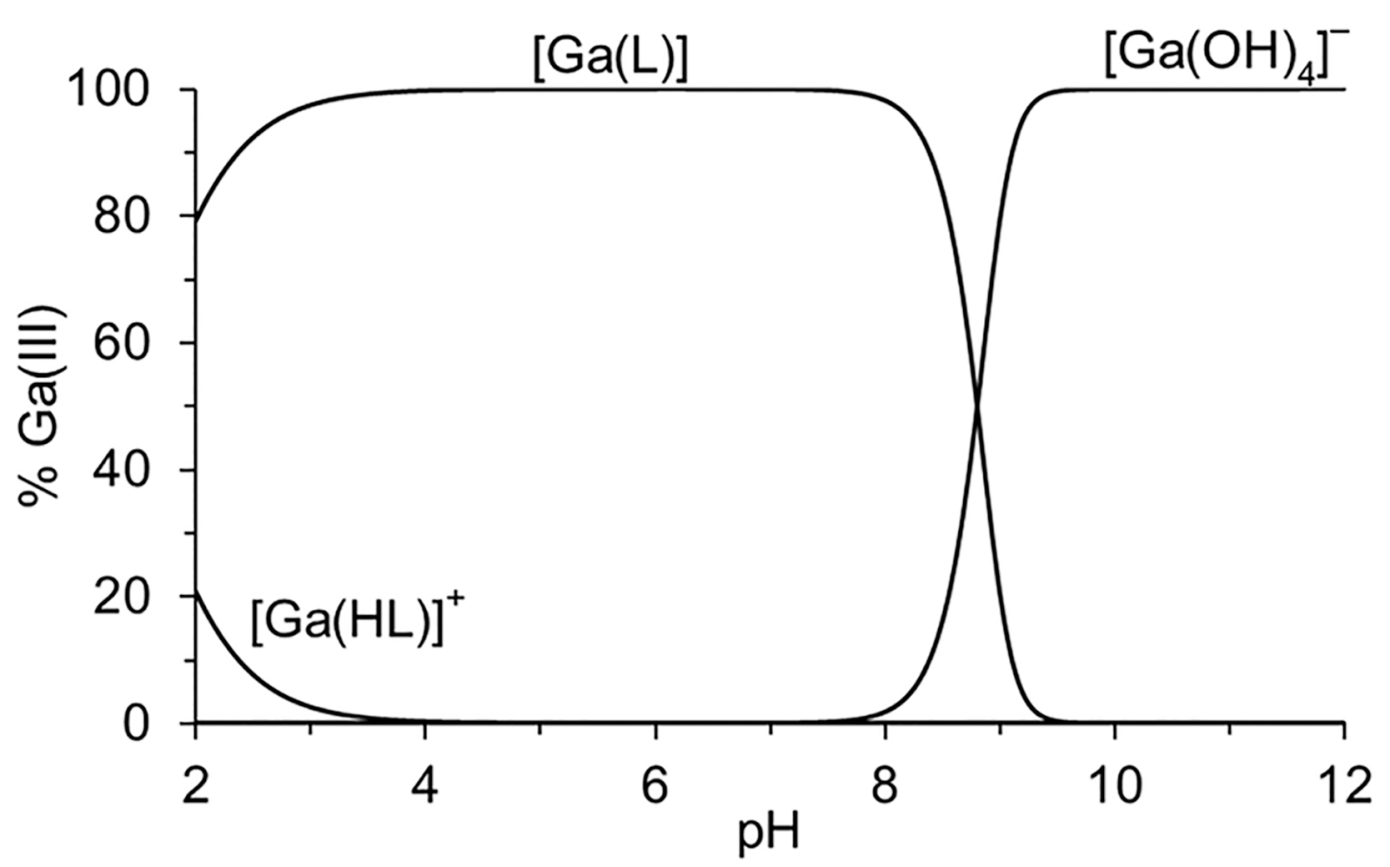
Distribution diagram of Ga(III)-**Tpaa** ([**Tpaa**] = [Ga] = 4 mM, 25 °C, and *I* = 0.1 M NMe_4_Cl).

The coordination properties of the **Tpaa** ligand toward Ga(III), Cu(II), and Zn(II) ions were studied in solution. Determination of the Zn(II) complex stability constant was disabled by extensive precipitation of the complex. Stability constants of the Ga(III)-**Tpaa** complexes (Table 2 and Tables S3 and S4) were determined by potentiometry. The out-of-cell method was used due to the long time required to reach equilibrium (competition between [Ga(**L**)] and tetrahydroxidogallate). Despite the low ligand basicity, the stability constant of [Ga(**L**)] is rather high and it is the dominant species along the whole acidic pH region (Figure 3). In addition, the protonated [Ga(**HL**)]^+^ species was identified in the strongly acidic region. As no free Ga(III) ion is present at the start of the titration, the stability constants were calculated from the equilibrium in the weakly alkaline region where the complex [Ga(**L**)] undergoes dissociation forming tetrahydroxidogallate at pH > 8. The absence of the mixed hydroxido complexes, [Ga(**L**)(OH)_*n*_]^*n*−^, in the chemical model is ascribed to their low abundance in solution. Some evidence of their formation can be seen through variable pH* ^1^H NMR (Figure S24). This result is in contrast to that previously reported for [Ga(**Dpaa**)(H_2_O)], where the mixed hydroxido species [Ga(**L**)(OH)]^−^ was the major species from pH 5–9.^22^ This corroborates the absence of a coordinated water molecule in the structure of [Ga(**Tpaa**)], as addition of a hydroxide to the Ga(III) coordination sphere would necessitate the removal of an N- or O-donor atom of a picolinate arm, ultimately leading to dissociation of the complex.

^1^H and ^71^Ga studies of the Ga-**Tpaa** system across a pH* range (Figures S24 and S25) reveals that some decomplexation can be seen in samples at pH* = 7.62 and higher, as evidenced by the formation of [Ga(OH)_4_]^−^ (δ_^71^Ga_ = 224, Figure S25) with increasing decomplexation under more basic conditions. The formation of free ligand can be seen in samples at pH* values of 8.97 and 10.07 (Figure S24). No evidence of the formation of Ga(OH)_3_ can be seen across the pH range studied. Note that the ^71^Ga signal corresponding to [Ga(**Tpaa**)] could not be observed due to the quadrupolar nature of ^71^Ga, making this peak very broad.

Variable temperature NMR of [Ga(**Tpaa**)] (D_2_O, pH* = 6.81, Figure S26) shows that with increasing temperature, no significant differences are observed in the ^1^H NMR. A minor change (0.05 ppm) is seen in the chemical shift of the CH_2_ protons when the sample is heated to 65 °C, but no change is observed for the protons corresponding to the picolinic acid arms. This indicates a high rigidity and kinetic inertness of the complex.

Comparison with **NOTA**, which is the gold standard for Ga(III) complexation, shows that the stability constants of **Tpaa** are several orders of magnitude lower (Table 2). However, one must consider that the ligand overall basicity (expressed as ∑(log *K*_1–4_)) is much lower than that of **NOTA** (∼16.5 vs ∼24, respectively). This indicates that the conditional stability constants might be comparable for the title ligand; indeed, pGa (calculated as −log[Ga(OH)_4_] ^–^ at a ligand concentration of 1 mM, a Ga(III) concentration of 0.1 mM, pH 7.4, and 25 °C) is 9.19 for [Ga(**Tpaa**)] and 11.82 for [Ga(**NOTA**)] (Table 2).

Stability constants of the Cu(II)-**Tpaa** complexes (Tables S3 and S4) were determined through a combination of potentiometry and UV–vis spectroscopy. The high complex stability combined with the low ligand basicity result in the complex being fully formed even in strongly acidic solutions, and only ∼20% of free Cu(II) ions are present even at pH 0 (Figure S18). Therefore, the stability constant of the [Cu(**L**)] complex was determined by a UV–vis competition titration with **H**_**4**_**edta** (Figures S19 and S20) following equilibration overnight. The wavelength corresponding to the absorption band maximum of [Cu(**edta**)]^2–^ at 740 nm was chosen for the calculation. Potentiometry was performed under both equimolar conditions and with the metal ion in excess, and thus, dinuclear species were also found in the chemical model; however, the second metal ion is coordinated only weakly (Tables S3 and S4). Despite the low ligand basicity, stability constants of the mononuclear complexes are rather high. The presence of the dinuclear species indicates that several ligand donor groups remain noncoordinated in the mononuclear species; this is expected due to the high ligand denticity. This is also the reason protonated complexes are formed in the very acidic region (Table S4). However, the rigidity of the pendant arms probably does not allow complete saturation of the Cu(II) coordination sphere, as could be concluded from the presence of the mixed hydroxido species in the alkaline region.

### Radiolabeling Studies with ^68^Ga

**Figure 4:**
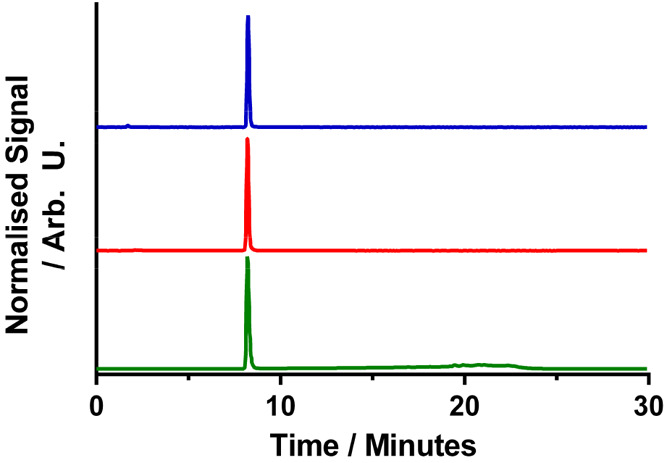
HPLC chromatograms of (blue, radiation detector) **Tpaa** labeled with ^68^Ga at pH 4.0 ([*L*] = 100 μM, *I* = 0.1 M sodium acetate buffer, *T* = 25 °C, *t* = 15 min), (red, radiation detector) **Tpaa** labeled with ^68^Ga at pH 7.4 ([*L*] = 100 μM, *I* = PBS, *T* = 37 °C, *t* = 15 min), (green, absorbance detector) [Ga(**Tpaa**)] standard. Note that the broad signal in the absorbance channel (15–25 min) is due to the absorbance of the solvent mixture changing across the gradient.

Radiolabeling of **Tpaa** with ^68^Ga was performed at pH 4.0 and 7.4. [^68^Ga][Ga(**Tpaa**)] was produced with a radiochemical conversion of >99% using 100 μM **Tpaa** and ^68^Ga in 15 min at both pH 4.0 (25 °C) and 7.4 (37 °C) (Figure 4). These results compare favorably to those of the standard chelators **DOTA** and **NOTA** and to other picolinate ligands for ^68^Ga, which are typically labeled at acidic pH, albeit at lower ligand concentrations (Table 1). Isolation of the radiolabeled species gave the product with a molar activity of 3.1 GBq/μmol. This is similar to the molar activity reported for [^68^Ga][Ga(**Dpaa**)(H_2_O)].^22^

**Figure 5:**
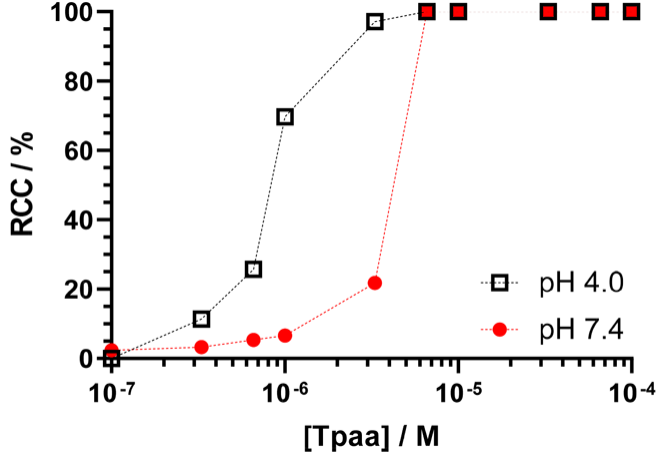
Effect of the ligand concentration on radiochemical conversion. Black open squares: **Tpaa** labeled with ^68^Ga at pH 4 (*I* = 0.1 M sodium acetate buffer, *T* = 25 °C, *t* = 15 min). Red filled circles: **Tpaa** labeled with ^68^Ga at pH 7.4 (*I* = PBS, *T* = 37 °C, *t* = 15 min). Dashed lines are for a guide to the eye.

The effect of ligand concentration on the radiolabeling efficiency of **Tpaa** was assessed at both pH 4.0 and 7.4 (Figure 5). At pH 4.0, **Tpaa** could be effectively radiolabeled at concentrations as low as 3.3 μM, comparable to the radiolabeling reported for **CHXdedpa** or **NOTA**.^26,28^ At pH 7.4, a concentration of 6.6 μM **Tpaa** was required for radiolabeling >99%, a significant improvement upon that reported for **Dpaa** and approaching the concentrations reported for **NOTA** under acidic conditions.^22,28^

When assessed for stability to 90% FBS, 32% of [^68^Ga][Ga(**Tpaa**)] was found to be intact after 30 min; however, after 2 h less than 5% of the radiolabeled complex remained intact (Figure S23). This is a significant improvement on the similar **Dpaa** picolinate system with ^68^Ga, which shows no stability to FBS after 30 min.^22^ However, this stability is ultimately insufficient for further development of this system as a ^68^Ga-radiotracer; other chelates show significantly higher stability to serum (e.g., [^67^Ga][Ga(**DOTA**)] retains 80% of ^67^Ga after 2 h and [^67^Ga][Ga(**NOTA**)] 98% after 2 h following incubation in 50% human serum),^24^ and this is a key requirement for the development of effective imaging agents.

## Conclusions

**Tpaa**, a tripodal picolinate-based chelator, has been synthesized in a shorter, five-step, route with an improved overall yield of 41%. **Tpaa** produces an octahedral complex upon coordination of Ga(III) despite having seven potential coordinating atoms. The [Ga(**Tpaa**)] system has an increased thermodynamic stability, with log *K*_GaL_ = 21.32, compared to that of [Ga(**Dpaa**)(H_2_O)], a similar system featuring a tripodal picolinate ligand with six potential coordinating atoms. Furthermore, there is no evidence of water coordination in the [Ga(**Tpaa**)] system when it is assessed by potentiometry or in the crystal structure, supporting the conclusion that the additional picolinate arm eliminates the coordinated water molecule seen in the [Ga(**Dpaa**)(H_2_O)] system.

**Tpaa** can be efficiently radiolabeled with ^68^Ga under mild conditions, with a >99% radiochemical conversion being achieved at pH 7.4 in PBS at a ligand concentration of 6.6 μM. The resulting radiolabeled complex, [^68^Ga][Ga(**Tpaa**)], has a molar activity of 3.1 GBq μmol^–1^. Serum stability was assessed using fetal bovine serum, with 32% of [^68^Ga][Ga(**Tpaa**)] remaining intact after incubation for 30 min, which is an improvement upon the previously reported [^68^Ga][Ga(**Dpaa**)(H_2_O)] that showed no stability under these conditions.

We have shown that for tripodal picolinate chelators, increasing the number of coordinating picolinate arms can improve the thermodynamic stability of the resulting Ga(III) complex, the radiolabeling properties with ^68^Ga, and the serum stability of the resulting ^68^Ga complex.

The versatility demonstrated by this chelator for the coordination of metals will likely lead to diverse applications in the future. These applications may depend upon the conjugation of **Tpaa** to small biomolecules, and such a bifunctional analogue should be developed. We envisage that the modification of a number of the picolinic acid arms into picolinic amides may be a successful route to develop mono- or multimeric targeted agents in the future.

## Supporting Information Available

Experimental details, synthetic methodology, potentiometric data, and crystal structure data (PDF)

## Abbreviations

^68^Ga: gallium-68
NMR: nuclear magnetic resonance
PET: positron emission tomography
FBS: fetal bovine serum
PBS: phosphate buffered saline
RCY: radiochemical yield

## Footnotes

a [Ga(Tpaa)]_2_·7H_2_O crystal structure data (CCDC Reference 1878045): refined formula C_42_H_44_Ga_2_N_8_O_19_; *M*_r_ = 1104.29; crystal dimensions 0.430 × 0.150 × 0.120 mm^3^; monoclinic; *P*2_1_; *a* = 11.5621(5) Å, *b* = 12.6405(4) Å, *c* = 15.2889(7) Å, α = 90°, β = 99.100(4)°, γ = 90°, *V* = 2206.36(16) Å^3^; *Z* = 2; ρ_calcd_ = 1.662 mg/m^3^; μ = 1.313 mm^-1^, λ = 0.71073 Å; *T* = 150(2) K; 2θ_max_ = 58.366°; no. of reflections measured (independent) = 23251 (9173); *R*_int_ = 0.0330; *R* = 0.0357 and *wR2* = 0.0483 (all data); ρ_max/min_ = 0.410/−0.359 e. Å^–3^; data collected using a Stoe IPDS2 diffractometer and processed using Stoe X-AREA and Sortav;^41^ structure solved using dual-space methods implemented within SHELXT^42^ and refined with SHELXL-2014.^43^
