# Supplementary material for: Inorganic Chemistry of the Tripodal Picolinate Ligand Tpaa with Gallium(III) and Radiolabeling with Gallium-68
Source: Inorg Chem. 2023 Oct 4;62(50):20769–76. doi: 10.1021/acs.inorgchem.3c02459 (PMC10731648; doi:10.1021/acs.inorgchem.3c02459)
Supplement: Supplementary file 1 — ic3c02459_si_001.pdf [file ic3c02459_si_001.pdf]

Inorganic chemistry of the tripodal picolinate ligand Tpaa with gallium(III) and radiolabeling with gallium-68.

Thomas W. Price,[a] Laurène Wagner,[a] Veronika Rosecker,[a] Jana Havlíčková,[b] Timothy J. Prior,[c] Vojtěch Kubíček,[b] Petr Hermann,[b] Graeme J. Stasiuk[a,]*

[a] Department of Imaging Chemistry and Biology, School of Biomedical Engineering and Imaging Sciences, King's College London, London, SE1 7EH, United Kingdom

[b] Department of Inorganic Chemistry, Faculty of Science, Charles University, Hlavova 8, 128 40 Prague 2, Czech Republic

[c] Chemistry, School of Natural Sciences, University of Hull, Cottingham Road, Hull, HU6 7RX, United Kingdom

* e-mail: graeme.stasiuk@kcl.ac.uk

Contents

1. *Materials and Methods* 1
2. *Potentiometric measurements* 2
3. *Radiolabelling* 3
4. *Synthetic procedures* 4
   4.1. *Synthesis of diethyl pyridine-2,6-dicarboxylate (**1**)* 4
   4.2. *Synthesis of ethyl 6-(hydroxymethyl)picolinate (**2**)* 6
   4.3. *Synthesis of ethyl 6-(chloromethyl)picolinate (**3**)* 8
   4.4. *Synthesis of triethyl 6,6',6''-(nitrilotris(methylene))tripicolinate (**4**)* 10
   4.5. *Synthesis of 6,6',6''-(nitrilotris(methylene))tripicolinic acid (**Tpaa**)* 12
   4.6. *Synthesis of (**Ga(Tpaa)**)* 15

5. Crystal Structure 20
6. Potentiometry Data 24
7. Radiochemistry Data 27
8. References 28

1. *Materials and Methods*

Unless otherwise stated, all chemicals were purchased from Sigma Aldrich (Dorset, United Kingdom). All solvents were purchased from VWR (Letterworth, United Kingdom). All commercially available starting materials were used without further purification.

NMR spectra were recorded on a JEOL ECP 400 MHz, JEOL Lambda 400 MHz or Bruker Avance 400 MHz spectrometer using the residual protic solvent signal as an internal reference. HRMS were recorded by the University of Hull mass spectrometry service on a Bruker Compass maXis impact spectrometer. pH measurements were carried out using a Jenway model 3520 pH/mV/temperature meter with a three point calibration.

2. *Potentiometric measurements*

Potentiometry was carried out according to previously published procedures.[1,2] Protonation and stability constants were determined in 0.1 M $(NMe_4)Cl$ at 25.0 °C with pK_w = 13.81.

Ligand protonation constants ([**Tpaa**] = 0.004 M) were determined from data obtained in pH range 1.7–12.1 (~40 points per titration and three parallel titrations).

The stability constants of the Ga(III) complexes ([**Tpaa**] = [Ga] = 0.004 M) were determined by the out-of-cell method in flame-sealed ampoules (equilibration time 4 weeks) as described previously (starting pH 1.5, ~15 points per titration and four parallel titrations). Points where $Ga(OH)_3$ precipitated following mixing were excluded as they were unable to reach thermodynamic equilibrium due to the low solubility and kinetic inertness of $Ga(OH)_3$.[3,4] Due to the complete formation of Ga^{3+}-**Tpaa** complex at acidic pH, the stability was assessed by competition with the tetrahydroxidogallate species, $[Ga(OH)_4]^-$, which forms in the alkaline region. The stability constants of the Ga^{3+}-hydroxido species were adopted from literature as follows: logβ(Ga(OH)) = 11.21, logβ($Ga(OH)_2$) = 21.72, logβ($Ga(OH)_3$) = 31.13, logβ($Ga(OH)_4$) = 38.64 (data corresponds to equilibrium: $Ga^{3+} + nH_2O \Leftrightarrow [Ga(OH)_n]^{3-n} + nH^+$).[5–7]

Stability constants of the Cu(II) complex ([**Tpaa**] = [Cu] = 0.004 M or [**Tpaa**] = 0.004 M, [Cu] = 0.008 M) were determined from data obtained in pH range 1.6–12.1, (50 data points per titration, three parallel titrations).

Please note that Zn(II) complex precipitated immediately after the reagent mixing and its stability constants cannot be determined.

The titration data were treated with OPIUM program package and the presented chemical models was chosen to have a chemical sense and exhibiting the best fitting statistics.[8,9] The calculated constants are concentration constants defined as $\beta_{hl} = [H_hL_l]/[H]^h\cdot[L]^l$ or $\beta_{hlm} = [H_hL_lM_m]/[H]^h\cdot[L]^l\cdot[M]^m$ and standard deviations are given directly by the program. Charges of the species in the text are omitted.

UV-Vis measurements: The UV-Vis spectra were recorded on spectrophotometer Specord 50 Plus (Analytik Jena AG). Temperature was maintained at 25.0 °C by Peltier block.

UV-Vis titration of Cu(II)-**Tpaa** system at pH range 0.0–1.7 ([**Tpaa**] = [Cu] = 0.004 M, pH was calculated from the added amount of HCl, without ionic strength control) was performed to confirm the chemical

model and these data were not used for determination of stability constant. The competition titration with H_4**edta** to determine the stability constant was performed at pH 7.45 maintained with 0.3 M HEPES buffer ([**edta**] = 0–16 mM, [**Tpaa**] = [Cu] = 0.004 M). These measurements were performed after equilibrating overnight at room temperature.

The following protonation constants of H_4**edta** and stability constant for the Cu(II)-H_4**edta** system were used: log$K$(HL) = 10.37, log$K$(H_2L) = 6.13, log$K$(H_3L) = 2.69, log$K$(H_4L) = 2.0, log$K$([Cu(L)]) = 18.78.[5–7]

The data from potentiometric titration and UV-Vis competition data were treated simultaneously with OPIUM program package.[8,9]

3. *pH* dependent NMR*

pH* measurements were carried out using a Jenway model 3520 pH/mV/temperature meter with a three-point calibration. pH* values are measured values in D_2O using a pH probe calibrated in H_2O.

Tpaa (63.7 µmol) was dissolved in D_2O (3 mL); to this was added 1.1 eq $GaCl_3$ in D_2O. The pH of the system was adjusted with DCl and NaOD. A fluffy white precipitate immediately formed.
After 48 hours, the samples were centrifuged to remove the precipitate, and the pH* recorded. These samples were transferred to NMR tubes and allowed to equilibrate for a further 24 hours. After 24 hours, no precipitate was observed. A capillary tube containing $Ga(NO_3)_3$ in D_2O was added, and the samples were analyzed by NMR.

4. *Radiolabelling*

Either:

A IGG100 generator was eluted with 0.6 M aq. HCl (4 mL). This eluate (200 – 300 MBq) was diluted with H_2O (20 mL).

Or

A GalliAd generator was eluted with 0.1 M aq HCl (1.1 mL). This eluate (450 MBq) was used without dilution.

The eluate was passed through a Strata-X-C 33 µM cation mixed-mode polymeric support. The column was washed with 0.1 M aq. HCl (2 x 1 mL). The activity was liberated from the column using 98:2 acetone:0.1 M aq. HCl (1.25 mL). Aliquots (20-30 MBq) of this solution were dried under a stream of inert gas at 90 °C and allowed to cool before use.

Ligand solution (1 mL) was added to the dried $^{68}GaCl_3$ and shaken. Aliquots (5 µL) were taken for analysis by TLC and aliquots (20 µL) for analysis by HPLC.

TLC analysis was performed on Kieselgel 60 F254 (Merck) or iTLC-SG (Agilent) plates with an eluate of 0.1 M citrate in water.

HPLC analysis was carried out using an Agilent Zorbax Eclipse XDB-C18 column (4.6 × 150 mm with 4.6 × 12.5 mm guard column) and a solvent system of water + 0.1% TFA and methanol (flow rate = 1 mL min^{-1}).

Gradient, [time/min](Solvent A (H_2O + 0.1% TFA):Solvent B (MeOH)): [0–3](95:5), [3–18](95:5–5:95), [18–20](5:95), [20–25](5:95–95:5), [25–30](95:5).

Radiolabelled products were isolated by semi-preparative HPLC using an Agilent Zorbax Eclipse XDB-C18 column (9.4 × 250 mm) and a solvent system of water + 0.1% TFA and methanol + 0.1% TFA (flow rate = 3 ml min^{-1}).

Gradient, [time/min](Solvent A (H_2O + 0.1% TFA):Solvent B (MeOH + 0.1% TFA)): [0–10](95:5), [10–11](95:5–5:95), [11–14](5:95), [14–15](5:95–95:5), [15–20](95:5).

Assessment of stability in foetal bovine serum:

Radiolabelling solution (100 µL) containing 100 µM ligand was added to foetal bovine serum (1.5 mL) and incubated at 37 °C. Aliquots were taken every 30 minutes for TLC analysis. ^{68}Ga incubated with serum moves up the TLC plate with an R_f of 0.5-1.0.[10]

5. *Synthetic procedures*

5.1. *Synthesis of diethyl pyridine-2,6-dicarboxylate (**1**)*

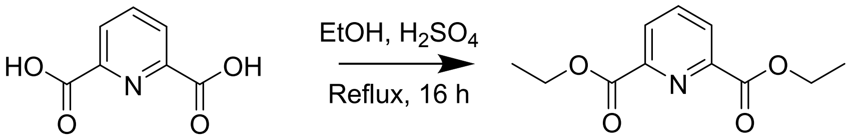

Pyridine-2,6-dicarboxylic acid (29.6 g, 177.1 mmol), ethanol (400 mL), and H_2SO_4 (100 µL, 1.87 mmol, 1 x 10^{-5} equiv.) were combined and the suspension heated to reflux overnight. The clear solution was cooled and concentrated to yield a colorless oil. The oil was resuspended in dichloromethane (100 mL) and washed with water (200 mL). The aqueous layer was extracted further with dichloromethane (3 x 100 mL). The combined organic layers were dried with Na_2SO_4, filtered, and concentrated to yield a clear oil that crystallized upon cooling (35.0 g, 156.8 mmol, 89%).

^{1}H NMR (400 MHz, $CDCl_3$, 298K), δ: 8.29 (d, 2H, **py**, $^3J_{HH}$ = 7.7 Hz), 8.01 (t, 1H, **py**, $^3J_{HH}$ = 7.7 Hz), 4.49 (q, 4H, OC$\mathbf{H_2}$CH$_3$, $^3J_{HH}$ = 7.1 Hz), 1.46 (t, 6 H, OCH$_2$C$\mathbf{H_3}$, $^3J_{HH}$ = 7.1 Hz)

^{13}C{^{1}H} NMR (100 MHz, $CDCl_3$, 298 K), δ: 164.64, 148.62, 138.20, 127.82, 62.36, 14.21

MS (ESI), *m/z*: 224.2 **[M+H]$^+$**

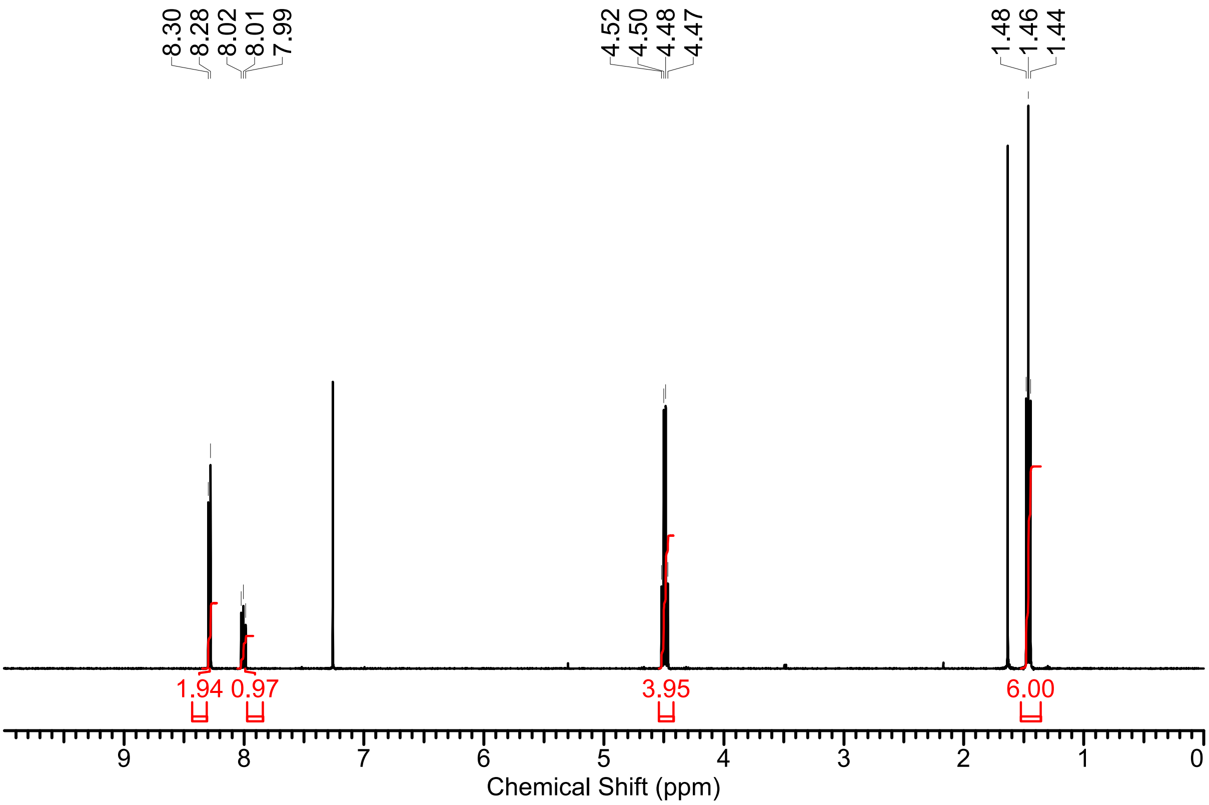

Figure S1: ^{1}H NMR of **1** ($CDCl_3$, 400 MHz, 298 K)

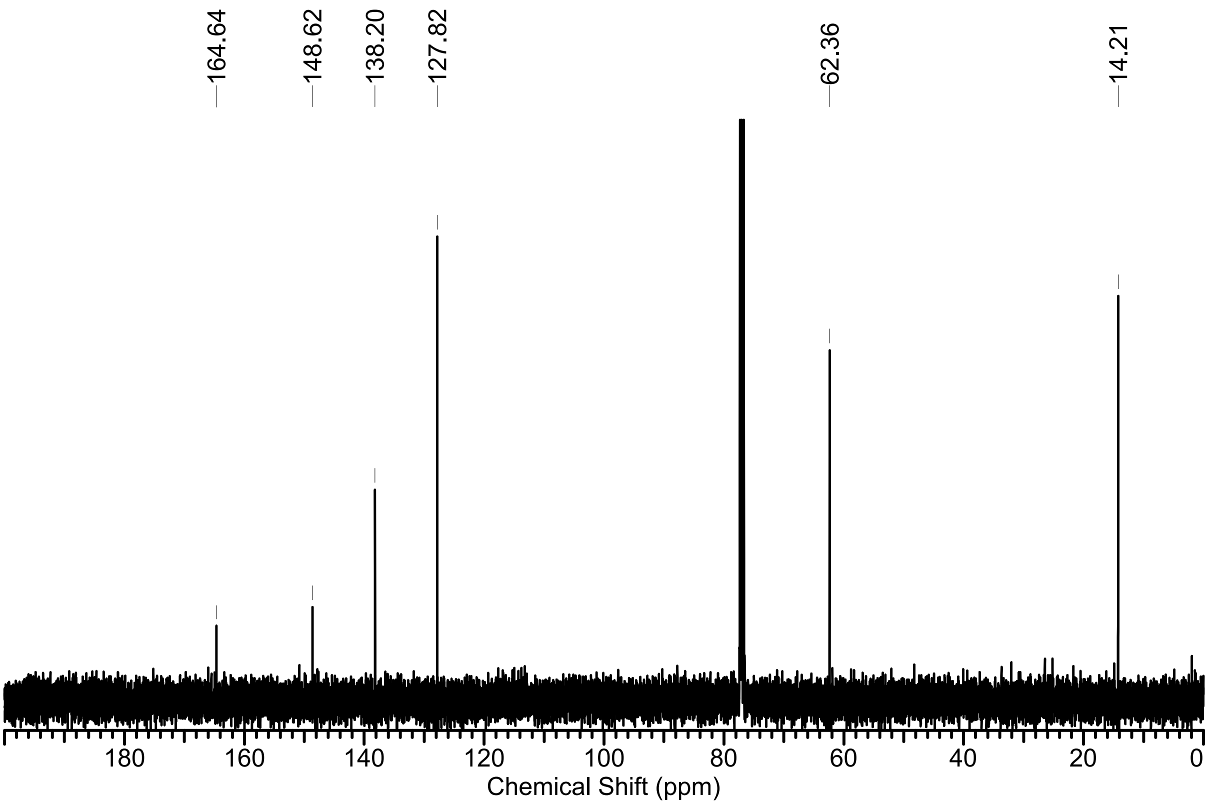

Figure S2: $^{13}C\{^{1}H\}$ NMR of **1** (100 MHz, $CDCl_3$, 298 K)

*5.2. Synthesis of ethyl 6-(hydroxymethyl)picolinate (**2**)*

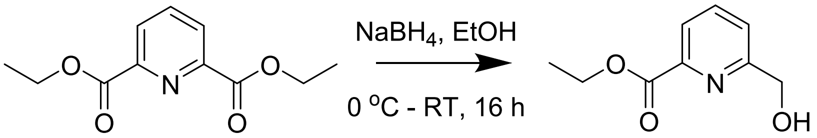

Compound **1** (31.0 g, 139 mmol, 1 equiv.) in ethanol (400 mL) was cooled in an ice bath. $NaBH_4$ (3.15 g, 83 mmol, 0.6 equiv.) was added portion-wise over 1 hour. The solution was allowed to warm to room temperature and stirred overnight. The solution was concentrated, and water (200 mL) was added. The solution was extracted with dichloromethane (5 x 100 mL) and the combined organic phases dried with $MgSO_4$, and the solvent removed to yield a crude product. The crude product was resuspended in minimal dichloromethane (50 mL). Ethyl acetate (100 mL) was added and hexane (100 mL) layered on top. The solution was stored in a freezer overnight, and the white crystalline product was collected by filtration and washed with hexane before drying to yield a white solid (10.7 g, 59 mmol, 42%).

^{1}H NMR (400 MHz, $CDCl_3$, 298 K), δ: 7.91 (dd, 1H, **py**, $^{3}J_{HH}$ = 7.8 Hz, $^{4}J_{HH}$ = 0.5 Hz), 7.75 (t, 1H, **py**, $^{3}J_{HH}$ = 7.8 Hz), 7.51 (dd, 1H, **py**, $^{3}J_{HH}$ = 7.8 Hz, $^{4}J_{HH}$ = 0.5 Hz), 4.80 (d, 2H, C**H_2**OH, $^{3}J_{HH}$ = 5.0 Hz), 4.48 (t, 1H, CH_2O**H**, $^{3}J_{HH}$ = 5.0 Hz), 4.37 (q, 2H, OC**H_2**CH_3, $^{3}J_{HH}$ = 7.2 Hz), 1.34 (t, 3H, OCH_2C**H_3**, $^{3}J_{HH}$ = 7.2 Hz)

$^{13}C\{^{1}H\}$ NMR (100 MHz, $CDCl_3$, 298 K), δ: 164.86, 160.59, 146.89, 137.39, 123.74, 123.31, 64.43, 61.66, 14.01

MS (ESI), *m/z*: 182.0 **[M+H]$^+$**

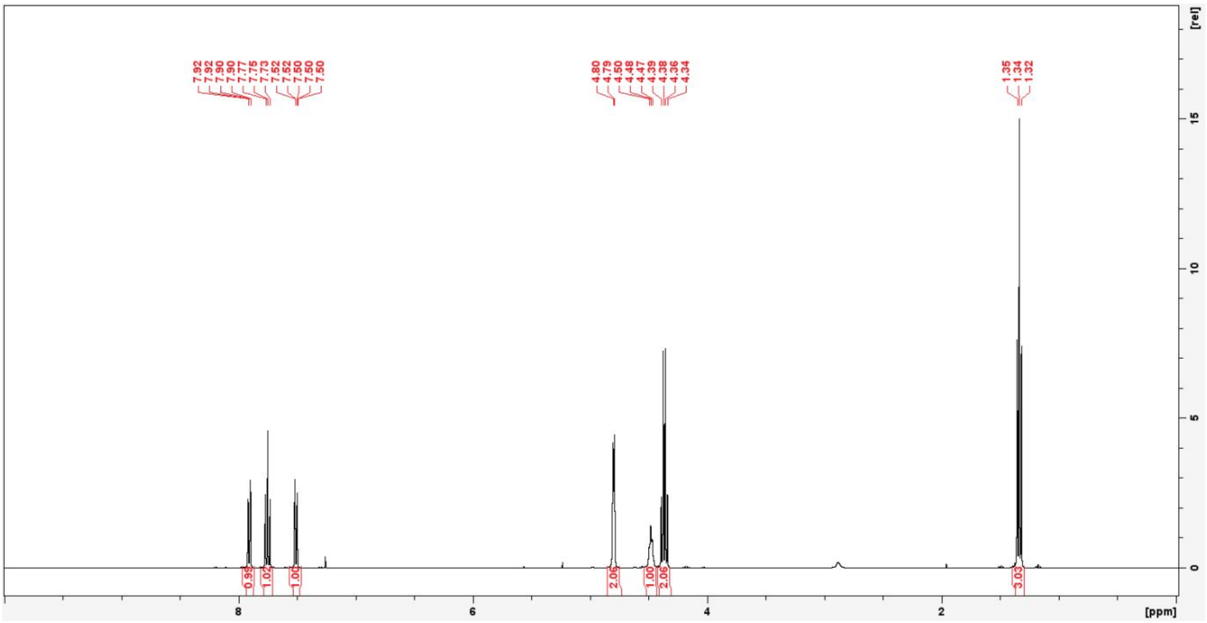

Figure S3: ^{1}H NMR of **2** ($CDCl_3$, 400 MHz, 298 K)

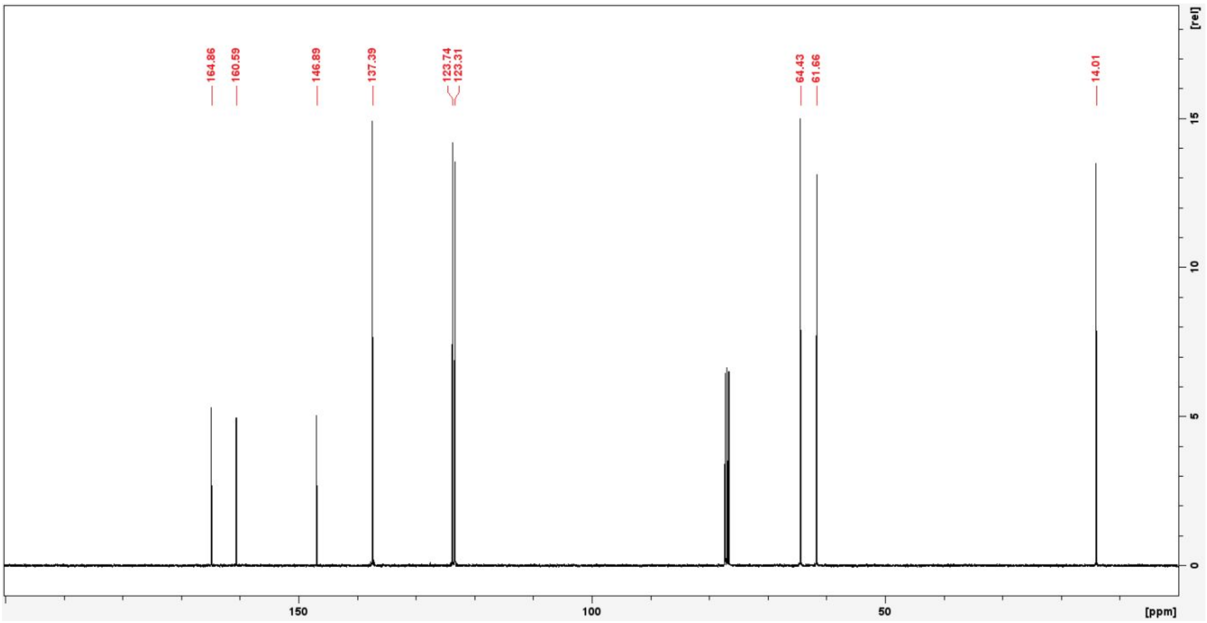

Figure S4: $^{13}C\{^{1}H\}$ NMR of **2** (100 MHz, $CDCl_3$, 298 K)

5.3. Synthesis of ethyl 6-(chloromethyl)picolinate (**3**)

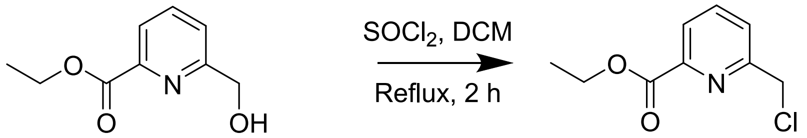

Compound **2** (5.74 g, 31.7 mmol, 1 equiv.) was dissolved in dichloromethane (230 mL), and thionyl chloride (4.6 mL, 63.4 mmol, 2 equiv.) was added slowly. The solution was heated to reflux for 2 hours before cooling and neutralized with saturated aq. $NaHCO_3$ solution. The organic layer was collected, and the aqueous layer was extracted with dichloromethane.(2 x 200 mL). The combined organic extracts were dried with $MgSO_4$, filtered, and concentrated to yield an orange oil (6.32 g, 31.7 mmol, 100%).

^{1}H NMR (400 MHz, $CDCl_3$, 298 K), δ: 7.99 (d, 1H, **py**, $^{3}J_{HH}$ = 7.8 Hz), 7.88 (t, 1H, **py**, $^{3}J_{HH}$ = 7.8 Hz), 7.69 (d, 1H, **py**, $^{3}J_{HH}$ = 7.8 Hz), 4.76 (s, 2H, C**H$_2$**Cl), 4.37 (q, 2H, OC**H$_2$**CH$_3$, $^{3}J_{HH}$ = 7.1 Hz), 1.32 (t, 3H, OCH$_2$C**H$_3$**, $^{3}J_{HH}$ = 7.2 Hz).

^{13}C{^{1}H} NMR (100 MHz, $CDCl_3$, 298 K), δ: 164.08, 156.95, 146.97, 139.11, 126.59, 124.61, 62.27, 45.59, 14.23

MS (ESI), *m/z*: 199.9 **[^{35}Cl][M+H]$^+$**, 201.9 **[^{37}Cl][M+H]$^+$**

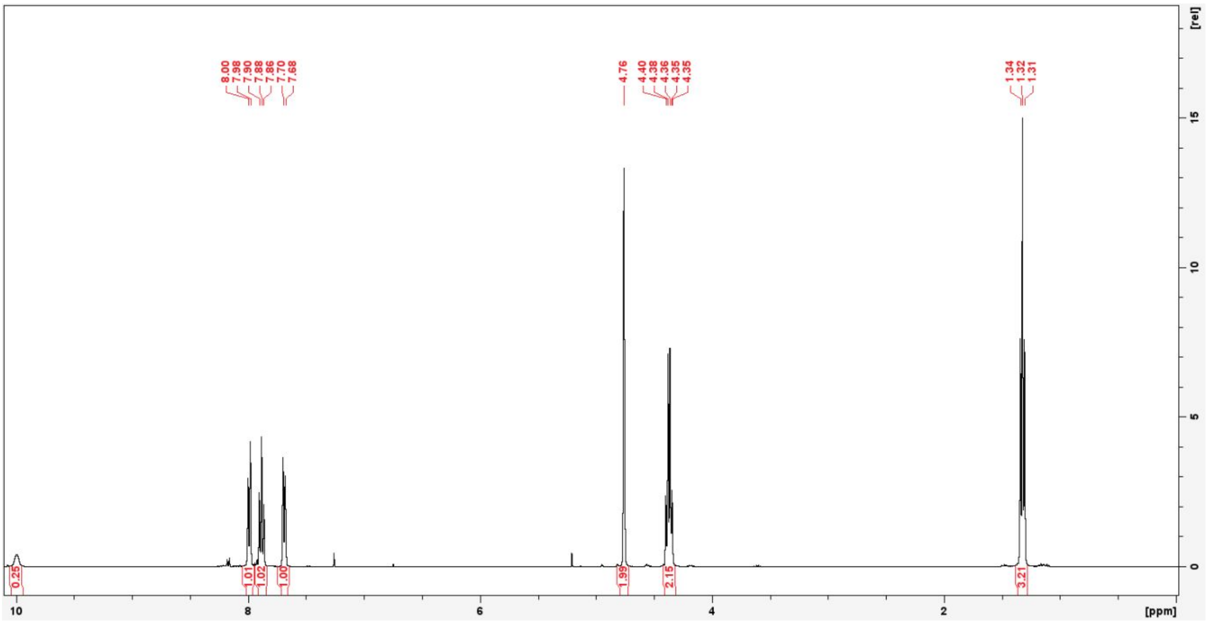

Figure S5: ^{1}H NMR of **3** (400 MHz, $CDCl_3$, 298 K)

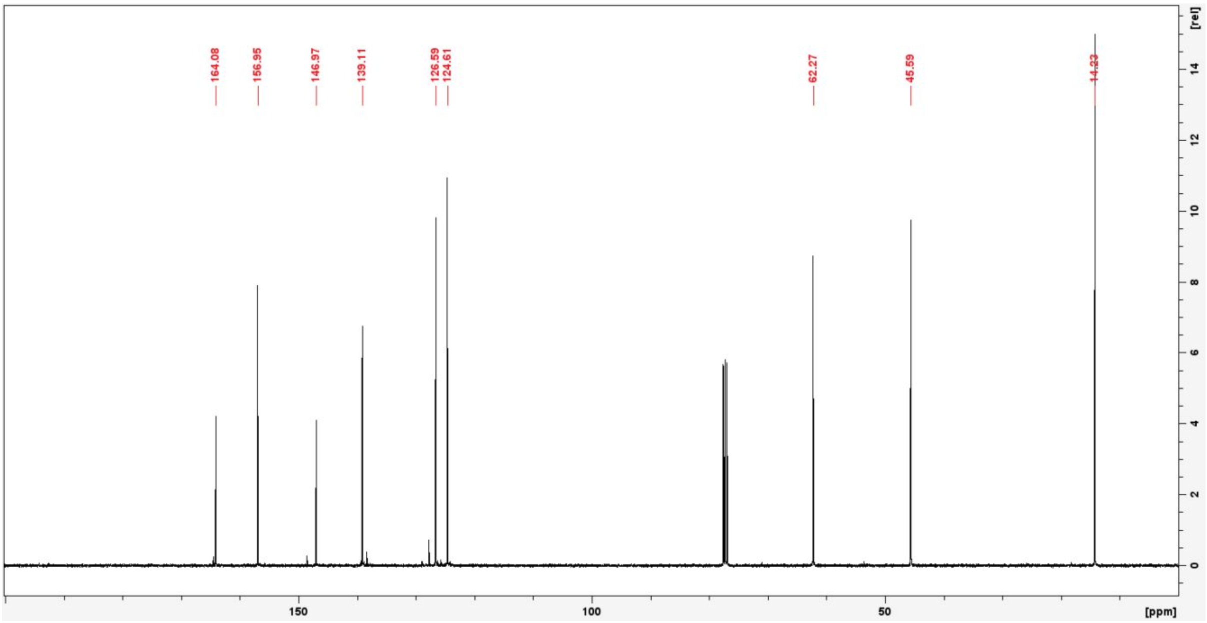

Figure S6: $^{13}C\{^{1}H\}$ NMR of **3** (100 MHz, $CDCl_3$, 298 K)

*5.4. Synthesis of triethyl 6,6',6''-[nitriletris(methylene)]tripicolinate (**4**)*

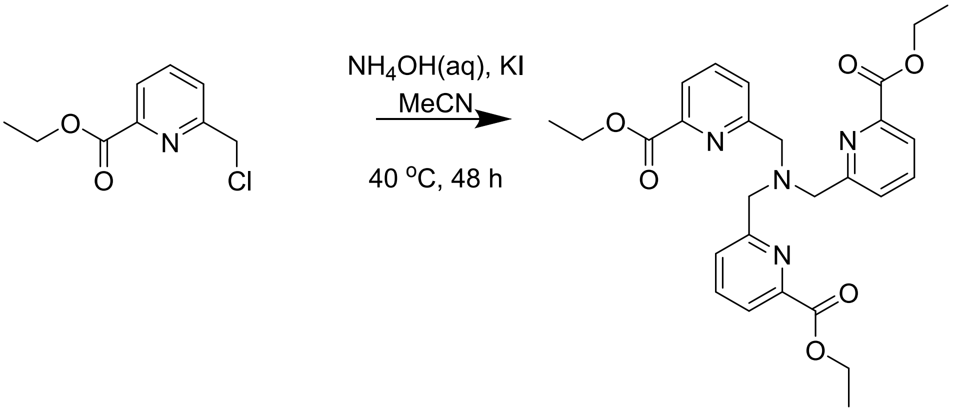

Aqueous ammonium hydroxide was heated under a stream of nitrogen; the exhaust fumes were passed through a calcium chloride drying tube before being bubbled through a solution containing **3** (1.64 g, 8.2 mmol,) in anhydrous acetonitrile (40 mL) at room temperature. After 2 hours, potassium iodide (135 mg, 0.8 mmol) was added and the solution heated to 40 °C overnight.

The solution was heated to 60 °C and further **3** (2.24 g, 11.2 mmol) was added portionwise over 6 hours and the solution heated overnight.

The reaction was quenched by addition of water (100 mL) and the solution was extracted with dichloromethane (3x100 mL). The combined organic extracts were dried with $MgSO_4$ and concentrated to yield a yellow oil. This was purified by automated flash chromatography to yield a yellow oil (2.36 g, 4.7 mmol, 73%).

^{1}H NMR (400 MHz, $CDCl_3$, 298 K), δ: 7.97 (dd, 3H, **py**, $^{3}J_{HH}$ = 7.7 Hz, $^{4}J_{HH}$ = 0.8 Hz), 7.87 (dd, 3H, **py**, $^{3}J_{HH}$ = 7.7 Hz, $^{4}J_{HH}$ = 0.8 Hz), 7.79 (t, 3H, **py**, $^{3}J_{HH}$ = 7.7 Hz), 4.45 (q, 6H, OC**H$_2$**CH$_3$, $^{3}J_{HH}$ = 7.1 Hz), 4.01 (s, 6H, C**H$_2$**N), 1.42 (t, 9H, OCH$_2$C**H$_3$**, $^{3}J_{HH}$ = 7.1 Hz)

^{13}C{^{1}H} NMR (100 MHz, $CDCl_3$, 298K), δ: 165.43, 159.89, 147.94, 137.43, 126.40, 123.66, 61.94, 59.79, 14.44

MS (ESI), *m/z*: 506.9 **[M+H]$^+$**

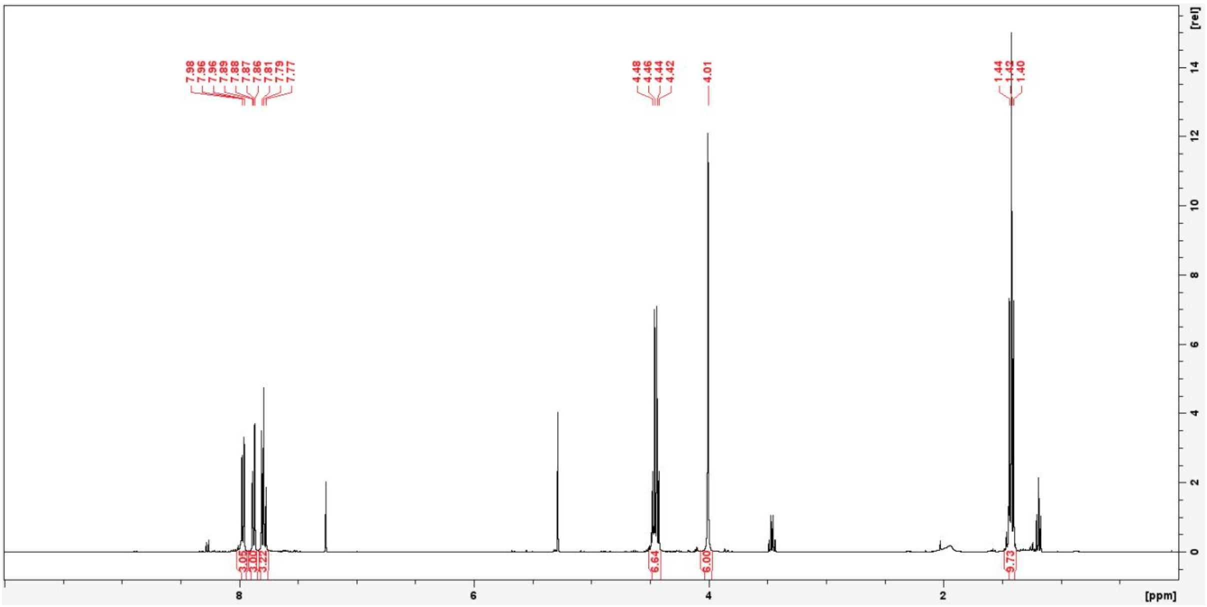

Figure S7: ^{1}H NMR of **4** (400 MHz, $CDCl_3$, 298 K)

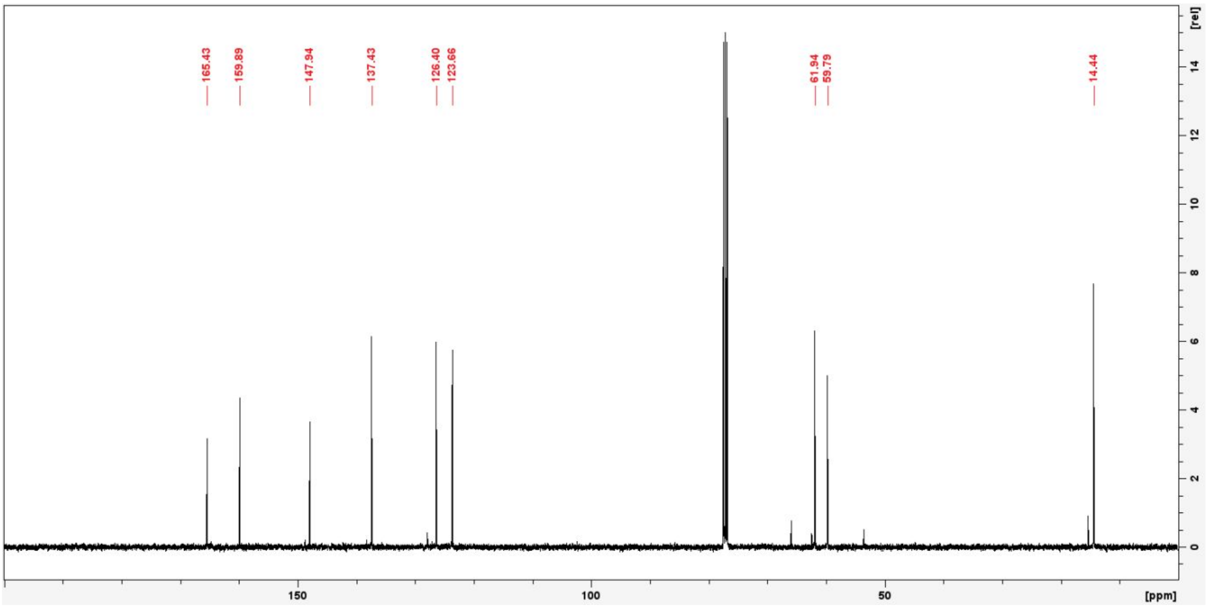

Figure S8: $^{13}C\{^{1}H\}$ NMR of **4** (100 MHz, $CDCl_3$, 298 K)

*5.5. Synthesis of 6,6',6''-[nitriletris(methylene)]tripicolinic acid (**Tpaa**)*

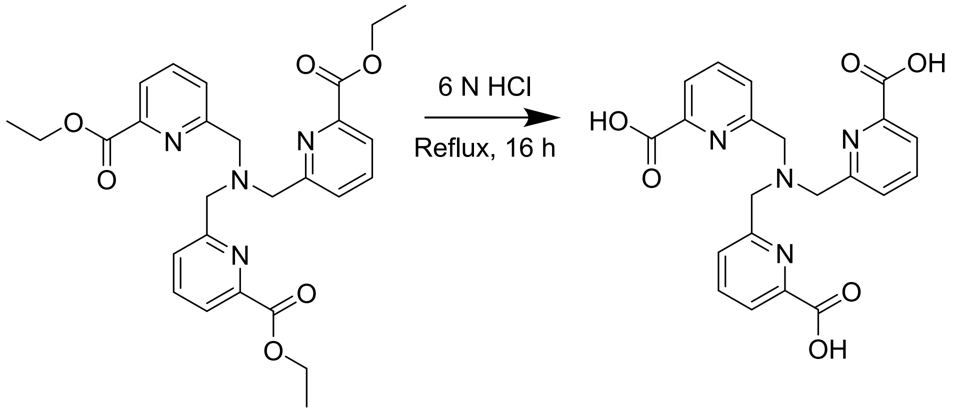

Compound **4** (2.2 g, 4.3 mmol) was dissolved in 6 M aq. HCl and the solution heated to reflux overnight. The solution was concentrated to dryness. The residue was resuspended in acetone and the precipitate collected and dried to yield an orange solid ((**Tpaa**)$(HCl)_{3.4}(H_2O)_{0.1}$, 2.3 g, 4.2 mmol, 97%).

^{1}H NMR (400 MHz, D_2O, 298 K), δ: 7.80-7.75 (m, 6 H, **py**), 7.28 (dd, 3 H, **py**, $^3J_{HH}$ = 6.0 Hz, $^4J_{HH}$ = 2.8 Hz), 3.65 (s, 6 H, pyC$\mathbf{H_2}$$NR_2$)

^{13}C{^{1}H} NMR (100 MHz, D_2O, 298 K), δ: 172.70, 157.83, 152.71, 138.30, 125.63, 122.39, 59.81

MS (ESI), *m/z*: 422.9 **[M+H]$^+$**

HRMS (ESI), m/z: 423.1298 (predicted for $C_{21}H_{19}N_4O_6$, **[M+H]$^+$**: 423.1299)

Elemental Analysis (C/H/N), *%*: 46.04/3.72/10.28 (predicted for $C_{21}H_{21.6}N_4O_{6.1}Cl_{3.4}$, (Tpaa)$(HCl)_{3.4}(H_2O)_{0.1}$: 46.01/3.97/10.22, M_w = 548.16 g mol^{-1})

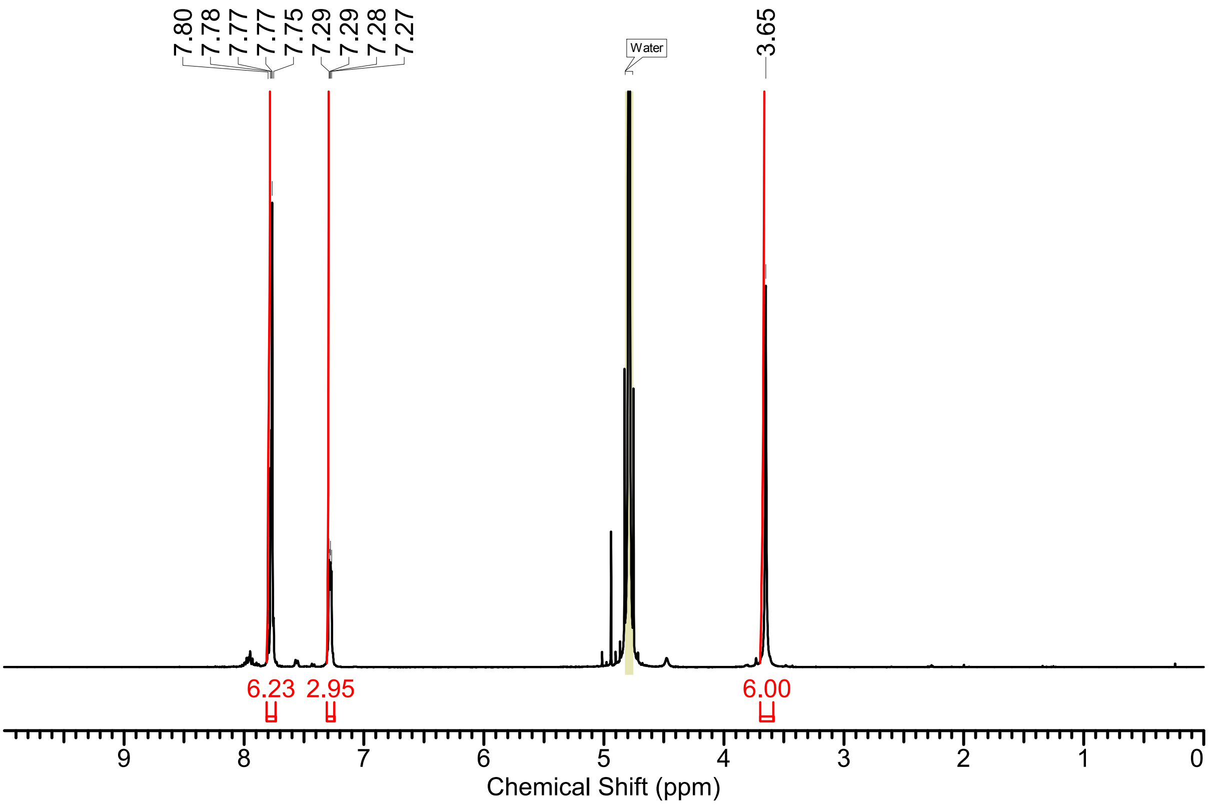

Figure S9: ^{1}H NMR of **Tpaa** (400 MHz, D_2O, 298 K)

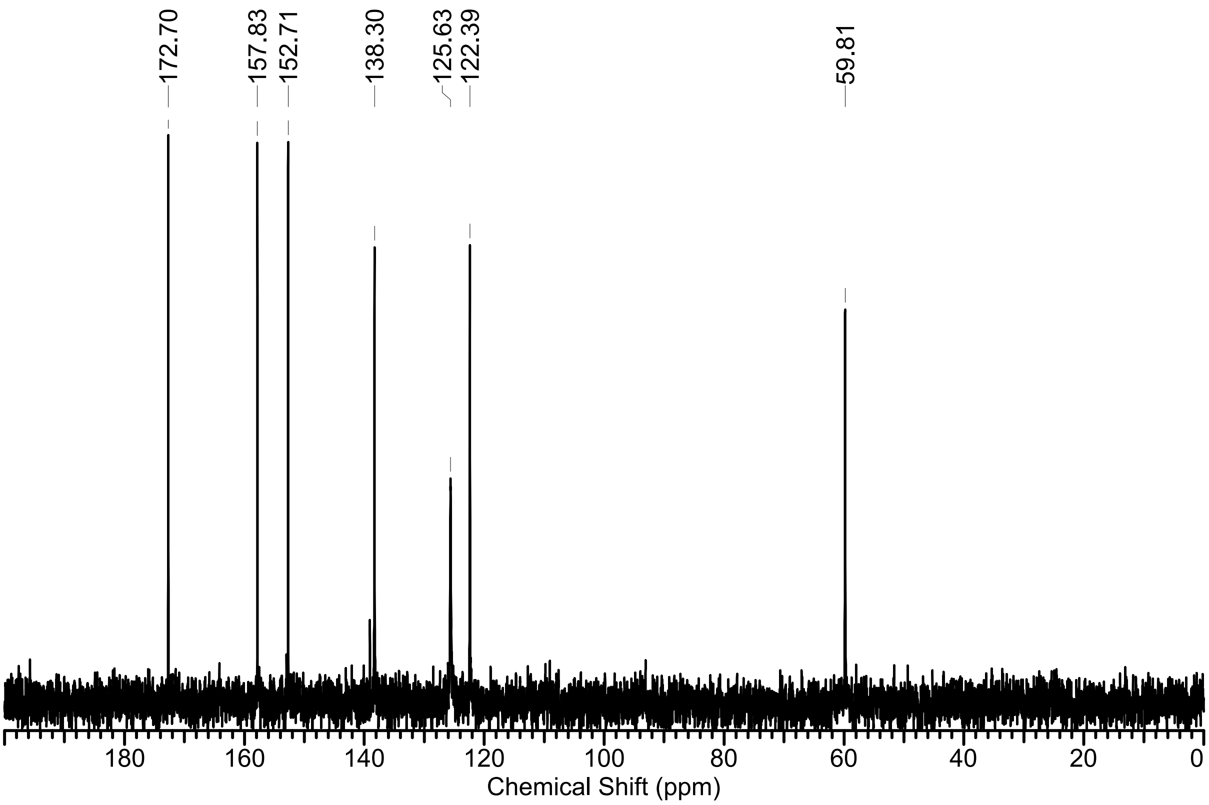

Figure S10: ^{13}C{^{1}H} NMR of **Tpaa** (100 MHz, D_2O, 298 K)

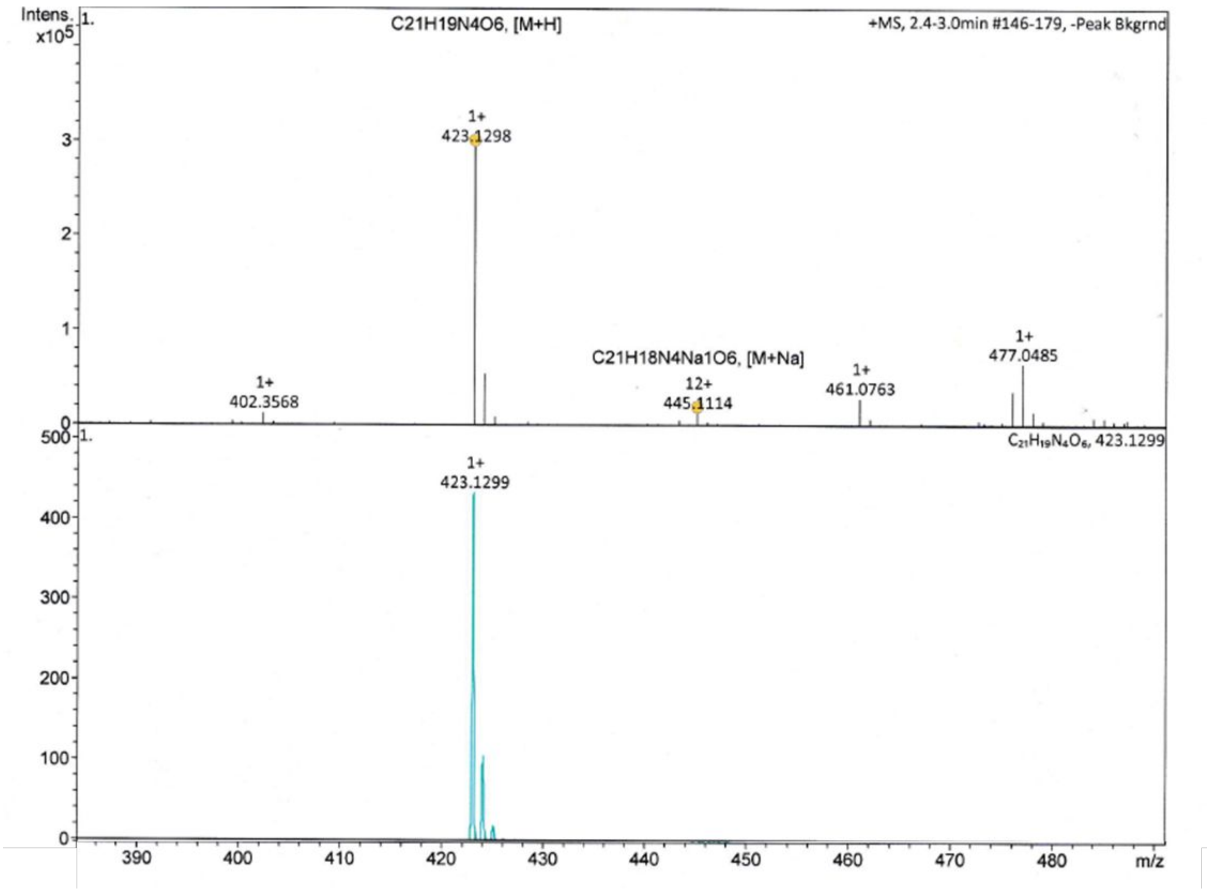

Figure S11: HRMS of **Tpaa**. Top: acquired data. Bottom: Simulated data for $C_{21}H_{19}N_4O_4$ **[M+H]**$^+$.

*5.6. Synthesis of [**Ga(Tpaa)**]*

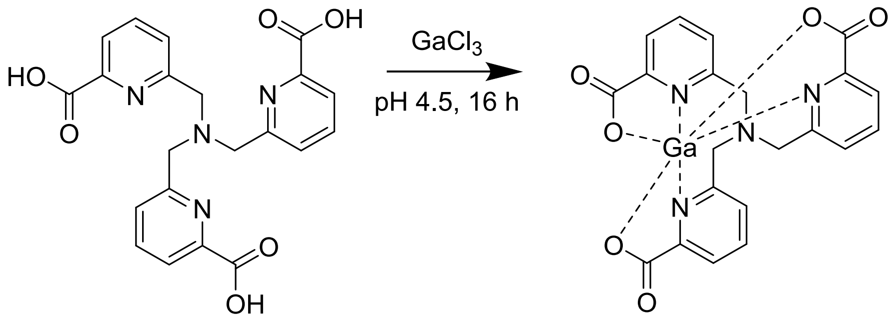

Tpaa (6.3 mg, 14.9 µmol, 1 equiv.) was dissolved in water (4.5 mL) and gallium chloride (2.6 mg, 14.7 µmol, 0.99 equiv.) was added. The pH was adjusted to 4.5 with sodium hydroxide and the solution was heated to reflux for 16 h. Drying yielded an off white solid which was used without further purification.

^{1}H NMR (400 MHz, D_2O, 298 K), δ: 8.42 (t, 3 H, **py**, $^{3}J_{HH}$ = 7.8 Hz), 8.33 (d, 3 H, **py**, $^{3}J_{HH}$ = 7.8 Hz), 7.93 (d, 3 H, **py**, $^{3}J_{HH}$ = 7.8 Hz), 4.56 (d, 3 H, pyC**H₂**NR₂, $^{2}J_{HH}$ = 17.4 Hz), 4.10 (d, 3H, pyC**H₂**NR₂, $^{2}J_{HH}$ = 17.4 Hz)

$^{13}C\{^{1}H\}$ NMR (100 MHz, D_2O, 298 K), δ: 167.01, 155.54, 145.73, 144.77, 128.93, 124.24, 53.31

MS (ESI), *m/z*: 488.7 **[^{69}Ga][M+H]$^+$**, 490.7 **[^{70}Ga][M+H]$^+$**

HRMS (ESI), *m/z*: 489.0321 (predicted for $C_{21}H_{16}{}^{69}GaN_4O_6$, **[M+H]$^+$**: 489.0320)

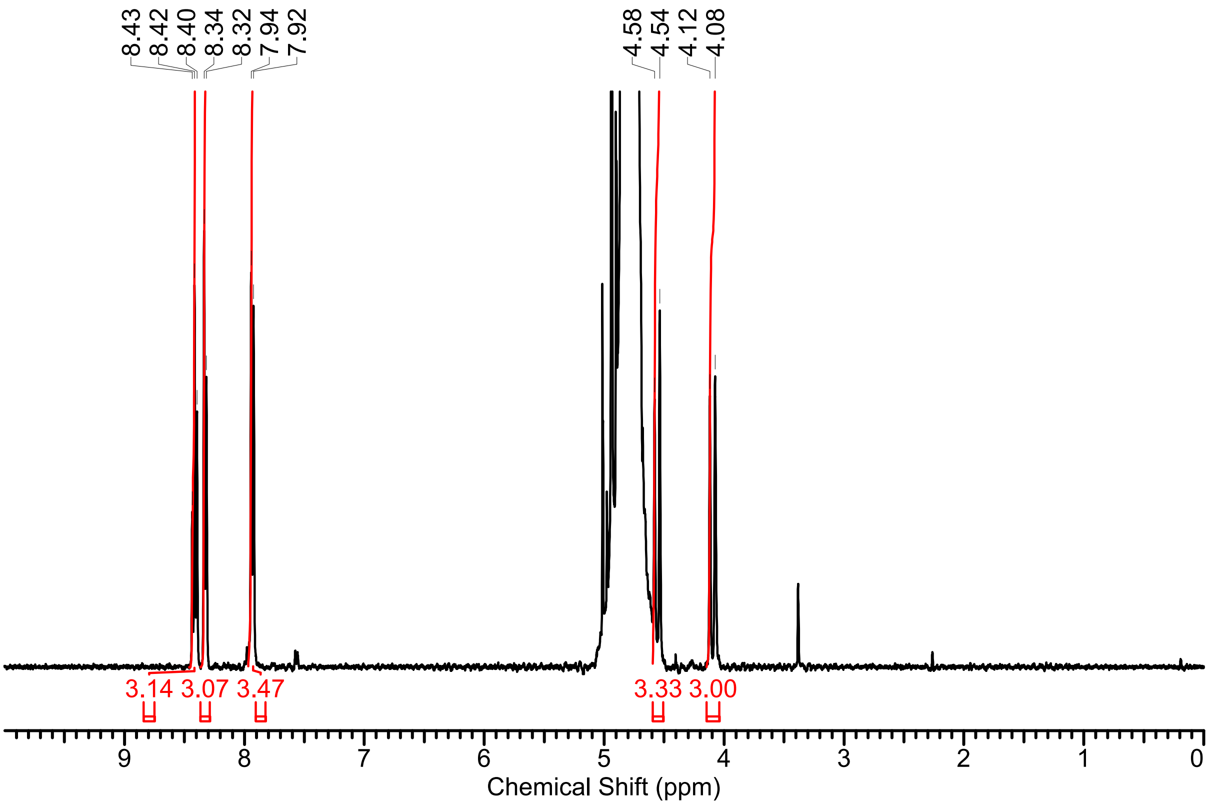

Figure S12: ^{1}H NMR of **[Ga(Tpaa)]** (400 MHz, D_2O, 298K)

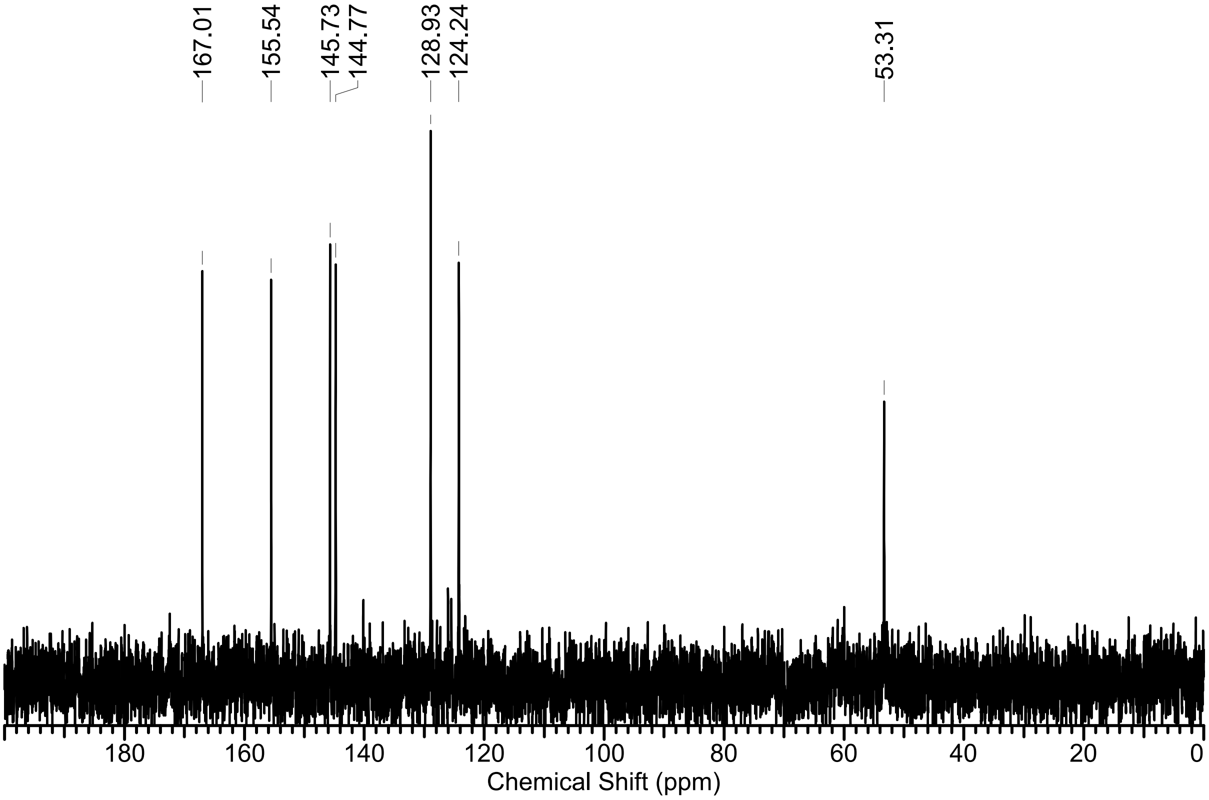

Figure S13: $^{13}C\{^{1}H\}$ NMR of **[Ga(Tpaa)]** (100 MHz, D_2O, 298 K)

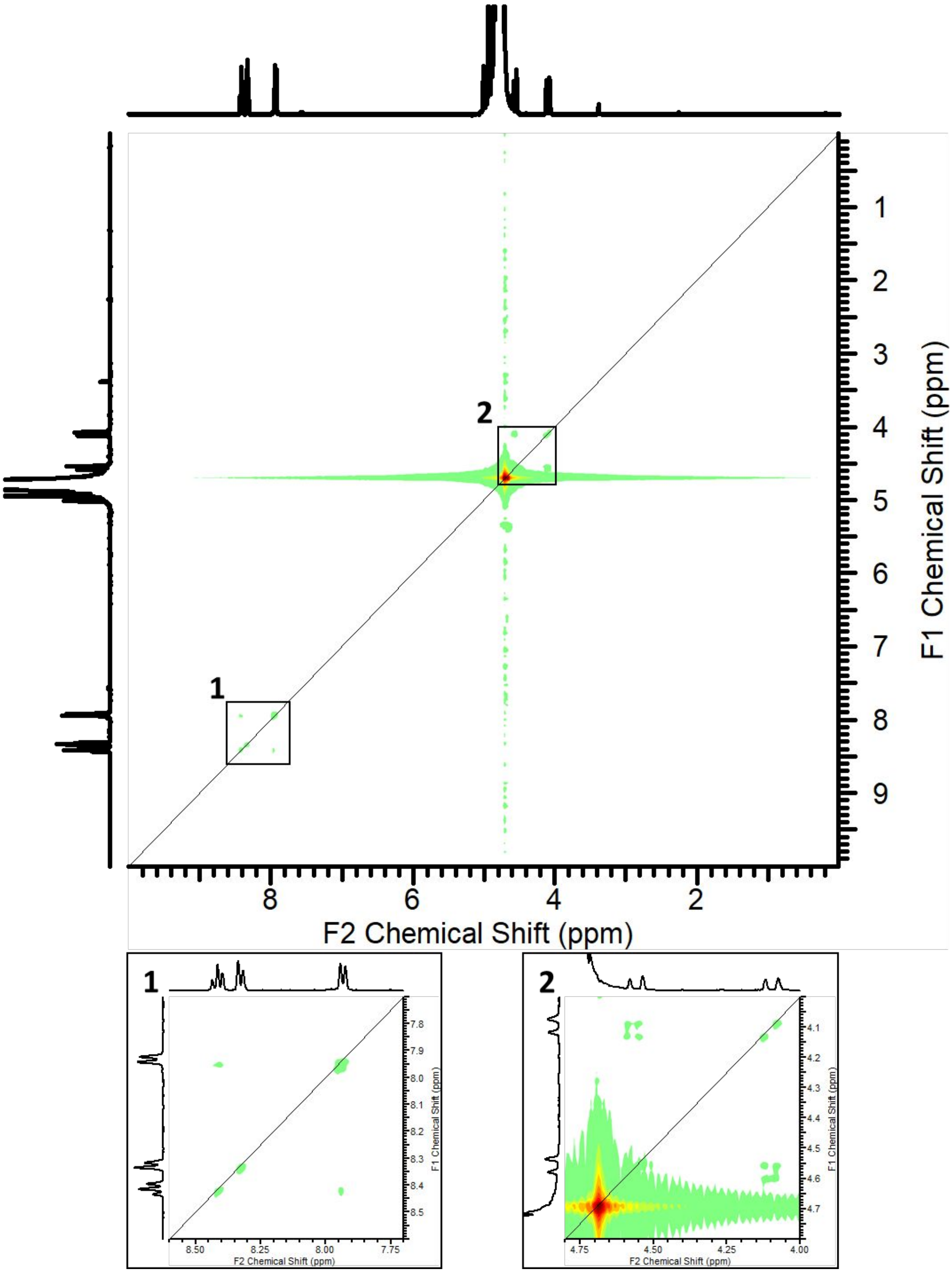

Figure S14: ^{1}H-^{1}H COSY of **[Ga(Tpaa)]** (400 MHz, D_2O, 298 K)

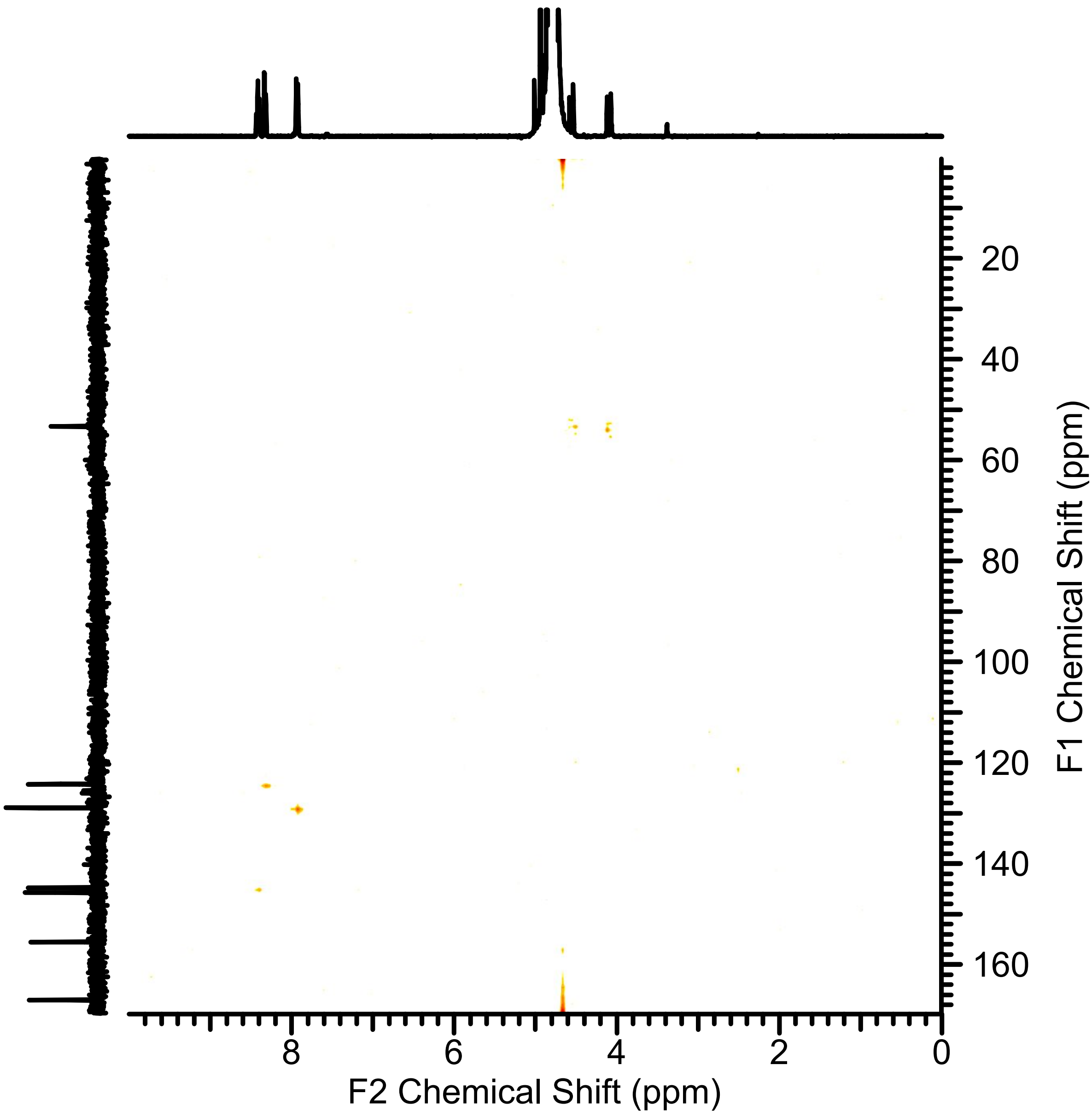

Figure S15: ^{1}H-^{13}C HMQC of **[Ga(Tpaa)]** (D_2O, 298 K)

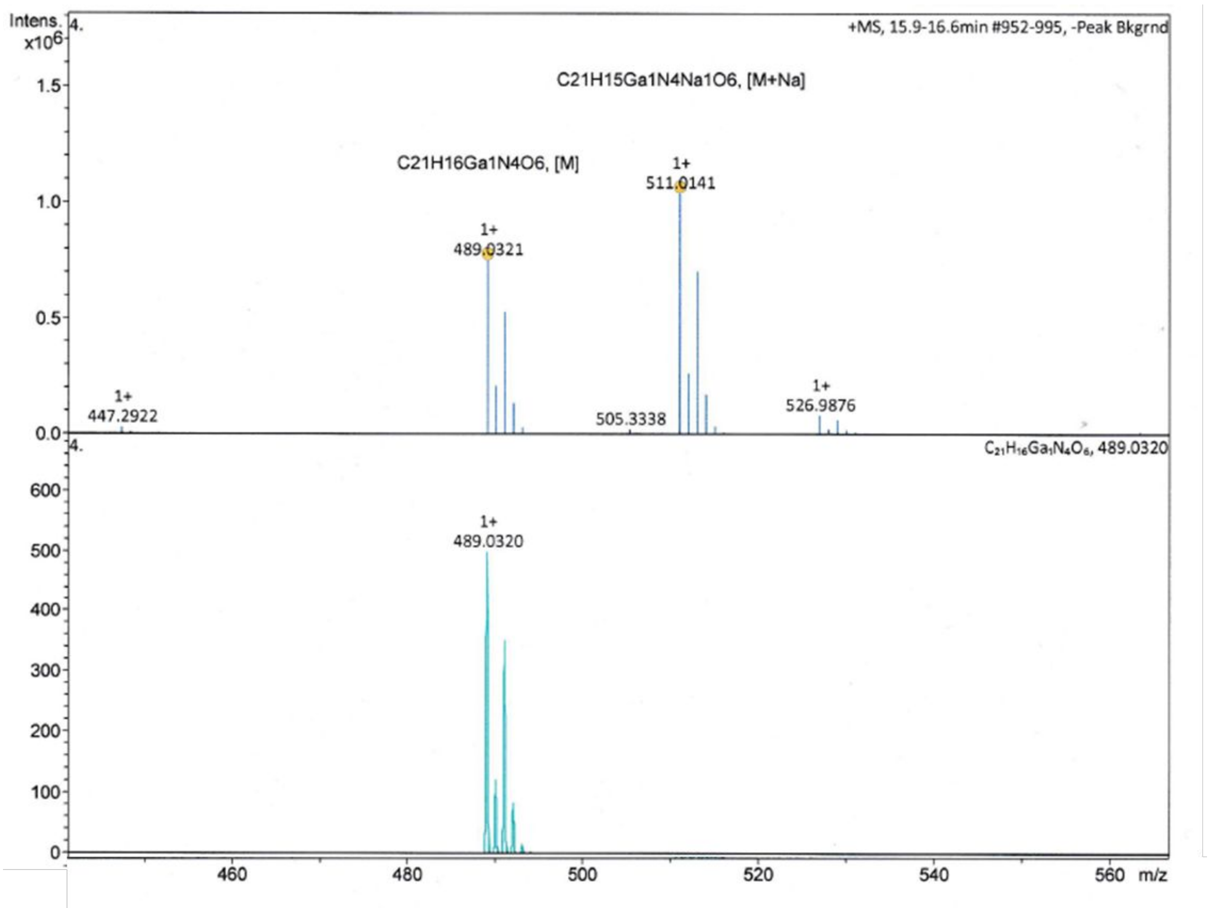

Figure S16: HRMS of **[Ga(Tpaa)]**. Top: acquired data. Bottom: Simulated data for $C_{21}H_{16}GaN_4O_4$ **[M+H]**$^+$.

6. Crystal Structure

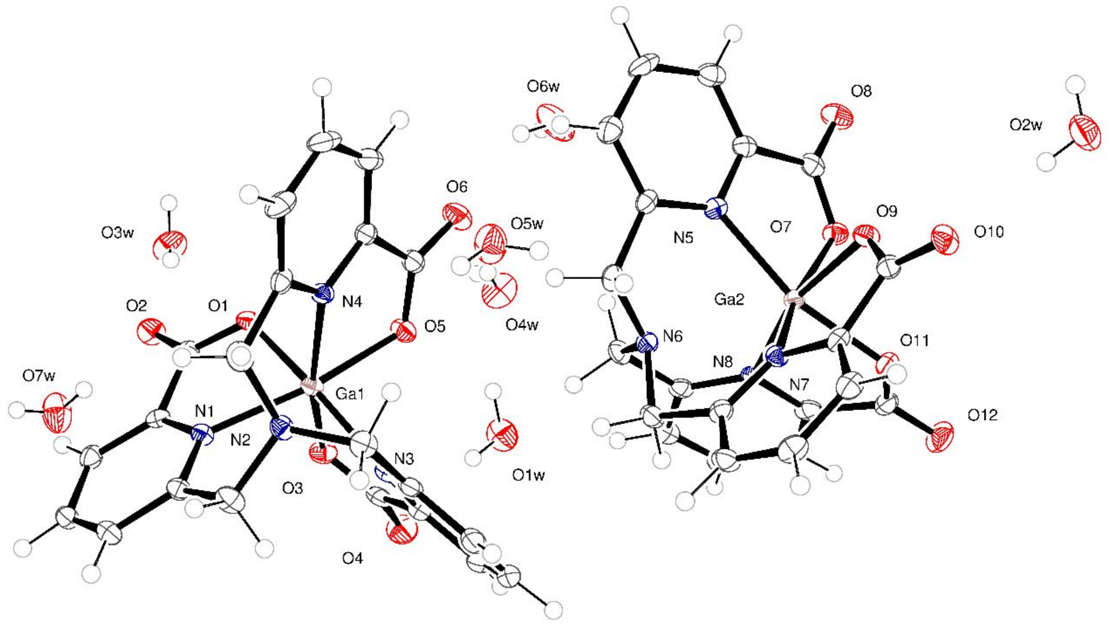

Figure S17: Crystal structure of **[Ga(Tpaa)]**$_2$·7H_2Oobtained from crystal grown at acidic pH.

The crystal structure was determined using routine methods. The asymmetric unit contains two Ga complexes and 7 molecules of water to give a composition of $[Ga(L)]_2.7H_20$ where L = $Tpaa^{3-}$. Each Ga^{3+} ion is surround by three chelating picolinates of the ligand. These are arranged in a *fac* alignment of the oxygen and nitrogen atoms of the chelates. The water molecules are crystallographically well resolved and form hydrogen-bonded clusters of seven water molecules. These clusters are enclosed within pockets in the structure and there are hydrogen bonds to the carboxylates of the ligand.

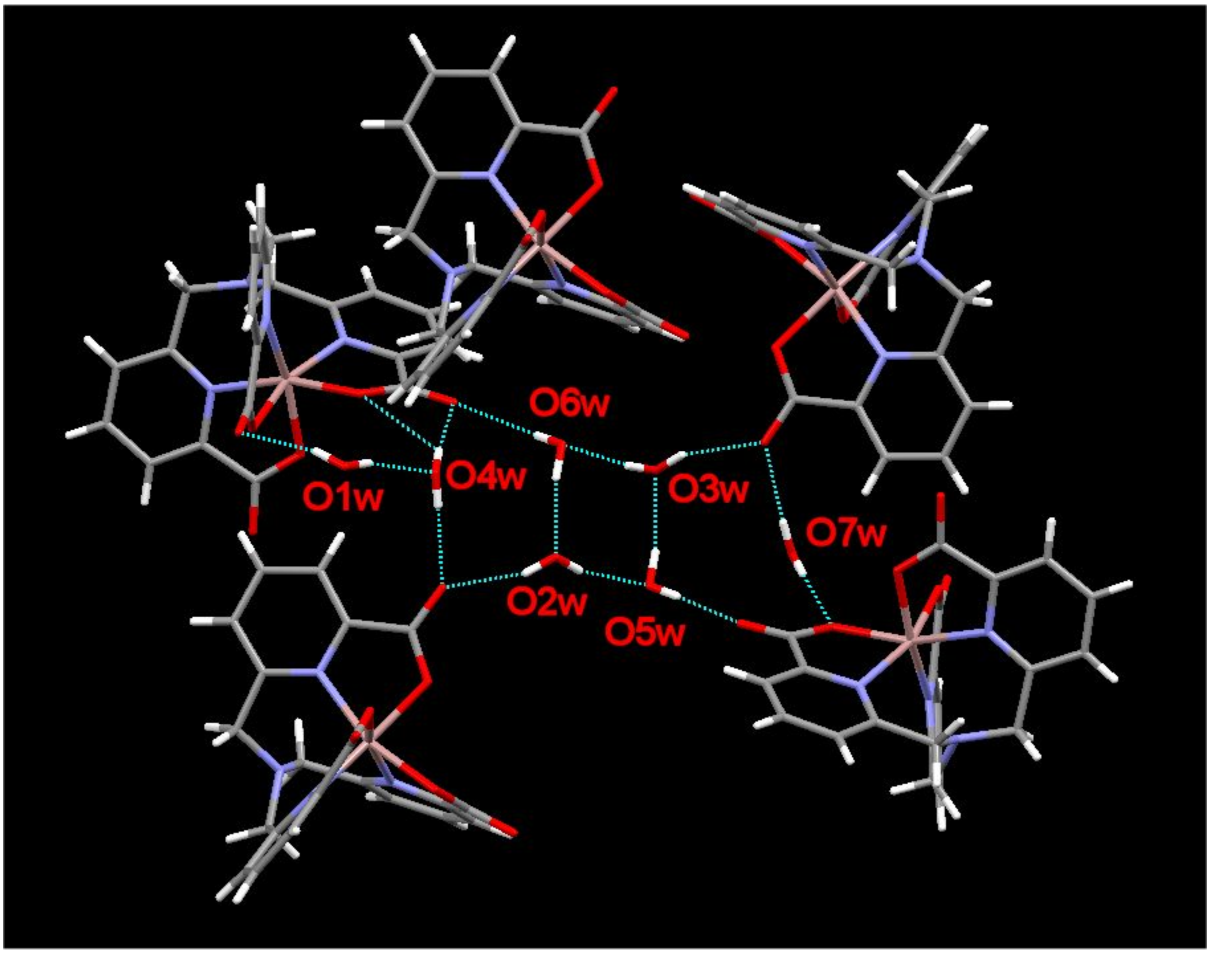

Figure S18: Cluster of 7 water molecules (note Ow1 breaks the pseudosymmetry)

The structure is pseudosymmetric; the two [Ga(L)$_3$] complexes within the asymmetric unit are related by an approximate (non-crystallographic) centre of symmetry. This is very close to being an exact centre of symmetry, but the water molecules do not display the same centre of inversion. There are very good reasons for believing that the non-centric P2$_1$ is the correct space group. Fundamentally, the structure will not solve in P2$_1$/m and there is no evidence in the intensity data for the presence of a glide plane to give space group P2$_1$/c for example. Perhaps more convincingly the refinement is well behaved, hydrogen atoms positions appear in Fourier difference maps, and the displacement parameters of all the atoms are reasonable. If this were a true centre of symmetry the refinement would be unstable and very likely the ellipsoids would show a problem. Note the quality of the fit to F-squared. wR(F^2) = 0.0483.

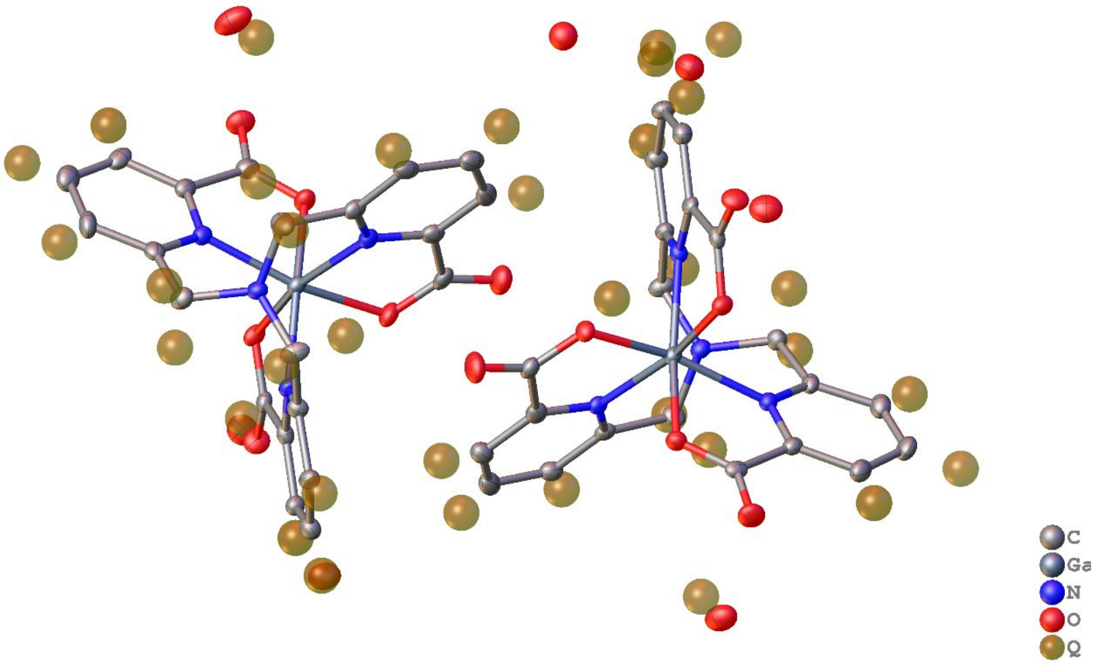

Figure S19: Representation of Fourier difference map calculated before hydrogen atoms are added to the model. Maxima in the 3-D electron density are shown as brown spheres, clearly highlighting the positions of hydrogen atoms.

Table S1: Crystal data and structure refinement for **[Ga(Tpaa)]**$_2$·7H_2O. CCDC Reference 1878045

Identification code	1878045	
Empirical formula	$C_{42}H_{44}Ga_2N_8O_{19}$	
Formula weight	1104.29	
Temperature	150(2) K	
Wavelength	0.71073 Å	
Crystal system	Monoclinic	
Space group	$P2_1$	
Unit cell dimensions	a = 11.5621(5) Å	α = 90°.
	b = 12.6405(4) Å	β= 99.100(4)°.
	c = 15.2889(7) Å	γ = 90°.
Volume	2206.36(16) Å^3	
Z	2	
Density (calculated)	1.662 Mg/m^3	
Absorption coefficient	1.313 mm^{-1}	
F(000)	1132	
Crystal size	0.430 x 0.150 x 0.120 mm^3	
Theta range for data collection	1.784 to 29.183°.	
Index ranges	$-15 \le h \le 15$, $-15 \le k \le 17$, $-14 \le l \le 20$	
Reflections collected	23251	
Independent reflections	9173 [R(int) = 0.0330]	
Completeness to theta = 25.242°	98.2 %	
Absorption correction	Semi-empirical from equivalents	
Max. and min. transmission	0.883 and 0.870	
Refinement method	Full-matrix least-squares on F^2	
Data / restraints / parameters	9173 / 24 / 684	
Goodness-of-fit on F^2	0.847	
Final R indices [I>2sigma(I)]	R_1 = 0.0269, $wR2$ = 0.0473	
R indices (all data)	R_1 = 0.0357, $wR2$ = 0.0483	
Absolute structure parameter	0.455(8)	
Extinction coefficient	none	
Largest diff. peak and hole	0.410 and –0.359 e.Å^{-3}	

7. *Potentiometry Data*

Table S2: Overall protonation constants of **Tpaa** (25 °C, *I* = 0.1 M NMe_4Cl).

$\log\beta_1$	6.95(1)
$\log\beta_2$	11.15(2)
$\log\beta_3$	14.51(2)
$\log\beta_4$	16.56(2)

Table S3: Overall stability constants, $\log\beta_{hlm}$, of the **Tpaa** complexes (25 °C, *I* = 0.1 M NMe_4Cl). Charges are omitted.

Species	Ga(III)	Cu(II)
[M(L)]	21.32(4)	16.39(5)
[M(HL)]	22.73(5)	19.37(5)
[M(H_2L)]	–	21.14(5)
[M(L)(OH)]	–	5.75(5)
[M_2(L)]	–	21.44(5)
[M_2(L)(OH)]	–	14.52(5)

Table S4: Stepwise stability constants log*K* of **Tpaa** complexes (25 °C, *I* = 0.1 M NMe_4Cl). Charges are omitted.

Equilibrium	Ga(III)	Cu(II)
M + L = [M(L)]	21.32	16.39
[M(HL)] = [M(L)] + H	1.41	2.98
[M(H_2L)] = [M(HL)] + H	–	1.77
[M(L)] + H_2O = [M(L)(OH)] + H	–	10.64
[M(L)] + M = [M_2(L)]	–	5.05
[M_2(L)] + H_2O = [M_2(L)(OH)] + H	–	6.92

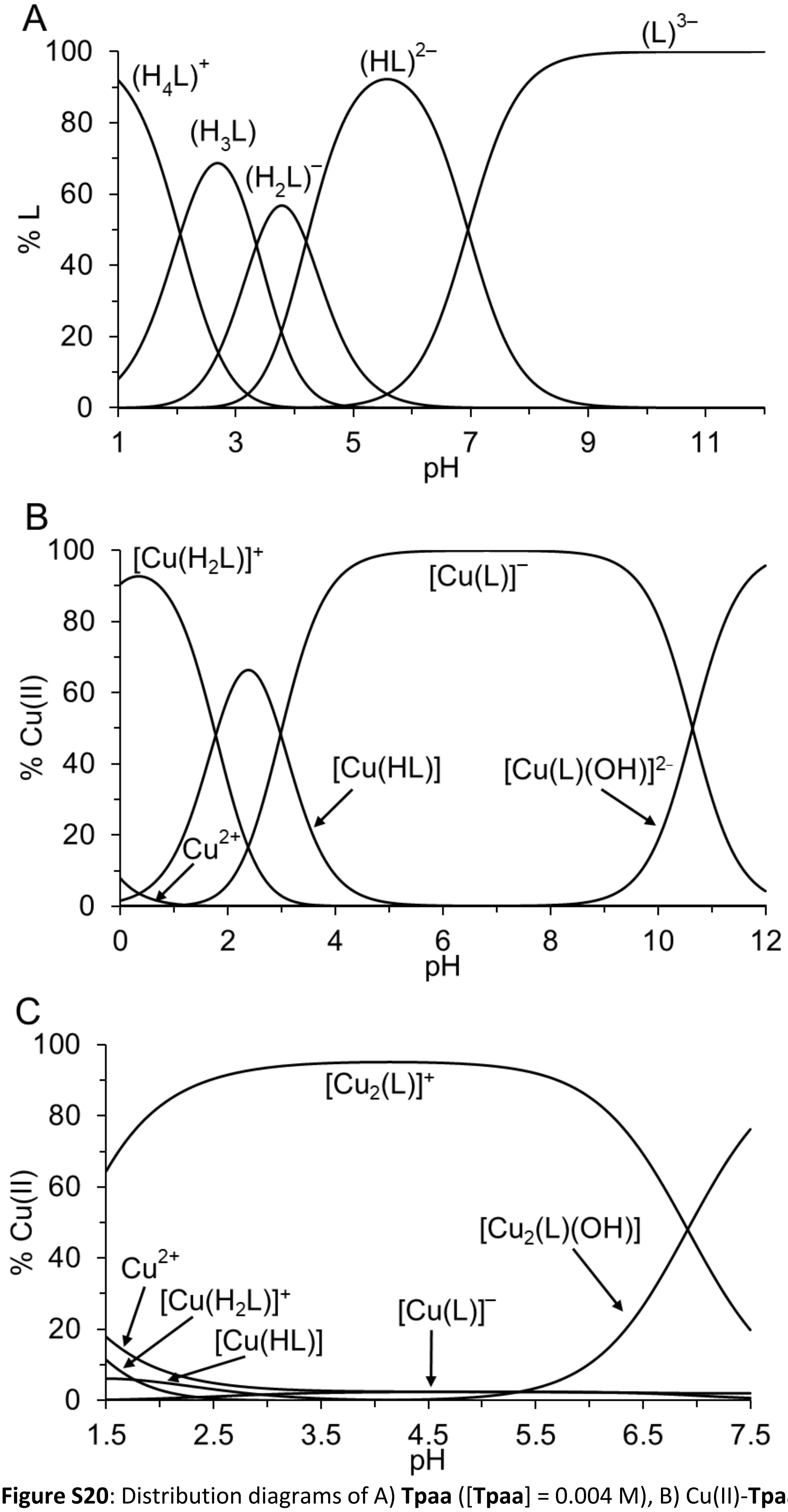

Figure S20: Distribution diagrams of A) **Tpaa** ([**Tpaa**] = 0.004 M), B) Cu(II)-**Tpaa** ([**Tpaa**] = [Cu] = 4 mᴍ), C) Cu(II)-**Tpaa** ([**Tpaa**] = 4 mM, [Cu] = 8 mᴍ) systems. (25 °C, *I* = 0.1 ᴍ NMe_4Cl).

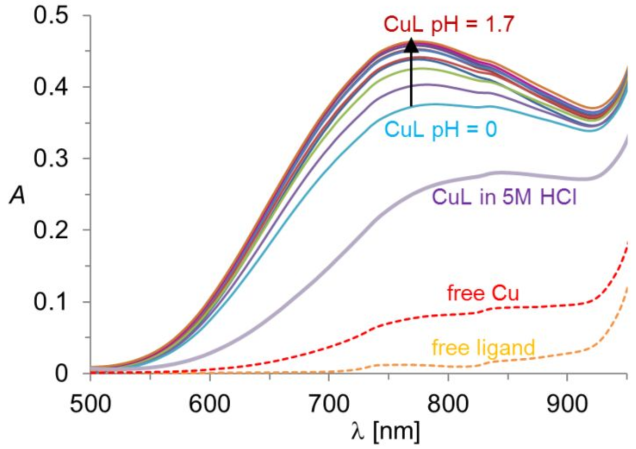

Figure S21: UV-Vis spectra of Cu(II)-**Tpaa** system in the strongly acidic region (25 °C, [**Tpaa**] = [Cu] = 4 mм).

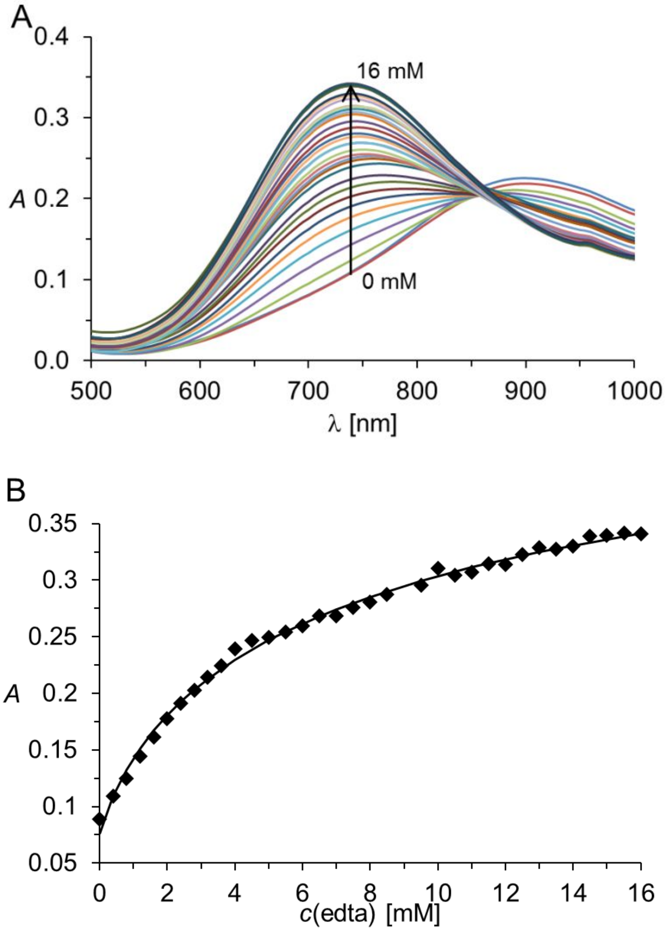

Figure S22: Competitive UV-Vis titration in the Cu(II)-**Tpaa**-H_4**edta** system. Spectra (A) and absorbance at 740 nm (B) as function of H_4**edta** concentration (pH 7.45, 25 °C, *I* = 0.1 м NMe_4Cl, [**Tpaa**] = [Cu] = 4 mм, [H_4**edta**] = 0–16 mм). The line represents the best fit.

8. Radiochemistry Data

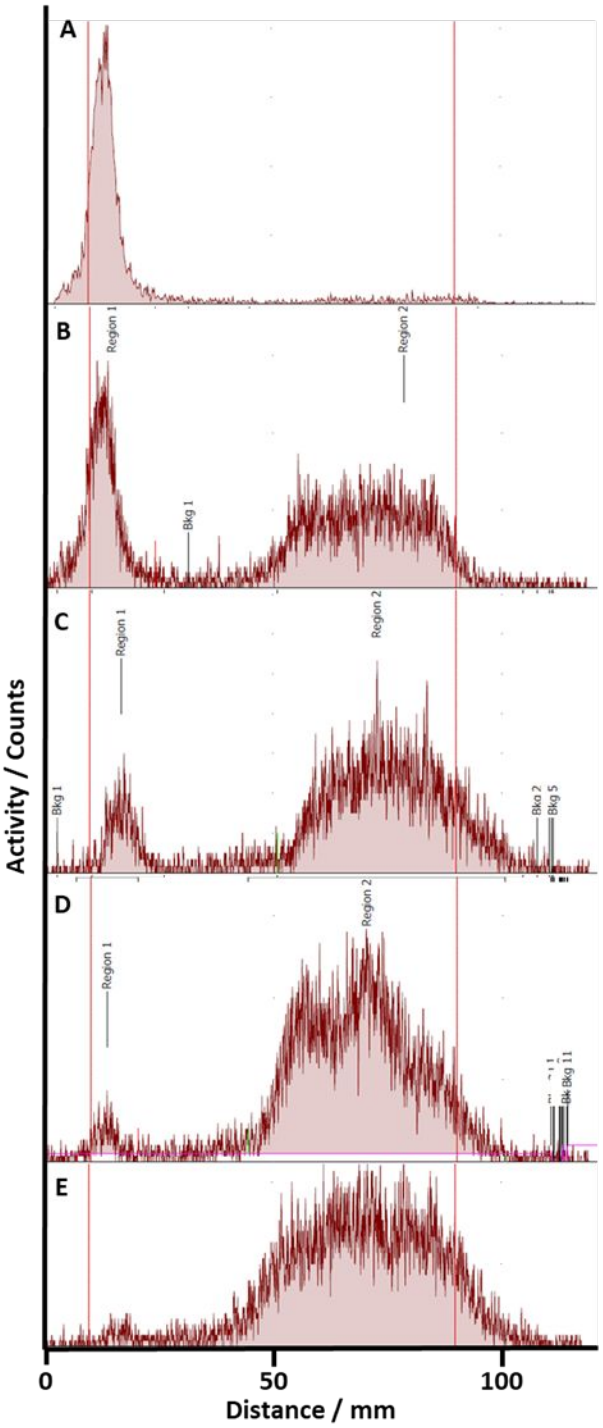

Figure S23: Serum stability of [^{68}Ga]**[Ga(Tpaa)]**. A) TLC of crude radiolabelling mixture (*[L]* = 100 µM, *I* = 0.1 M acetate, pH = 4.0, *T* = 25 °C, *t* = 15 min) B) TLC of solution after 30 min incubation with foetal bovine serum at 37 °C, C) 60 min incubation, D) 90 min incubation, E) 120 min incubation.

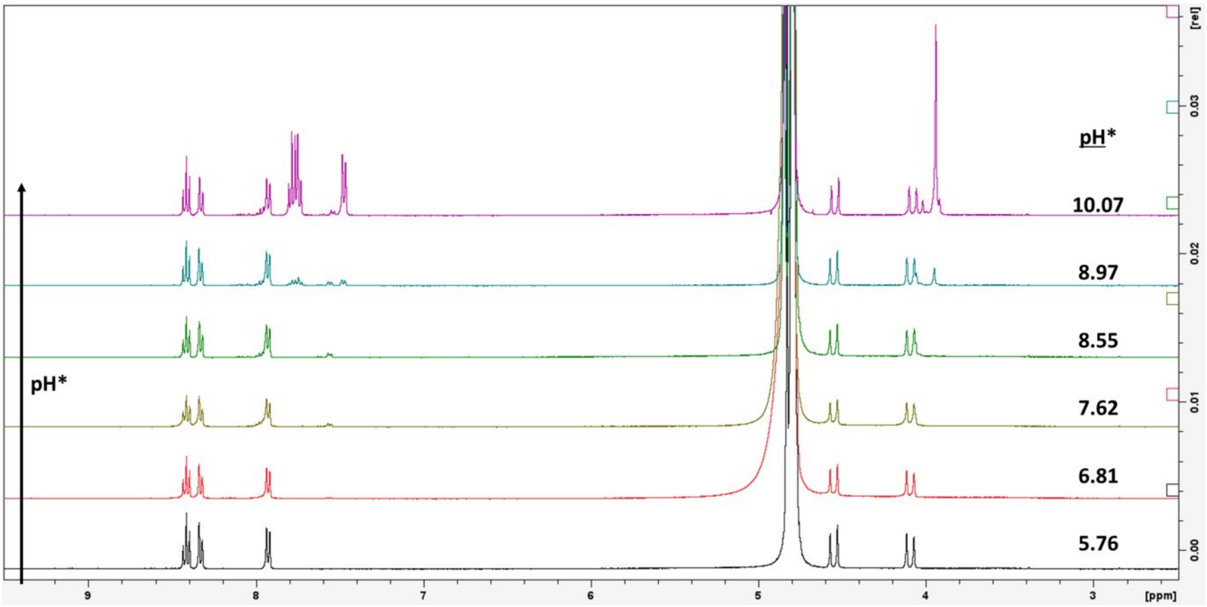

Figure S24: ^{1}H NMR of [Ga(Tpaa)] in D_2O at various pH* values. pH* values are indicated. (400 MHz, 298 K)

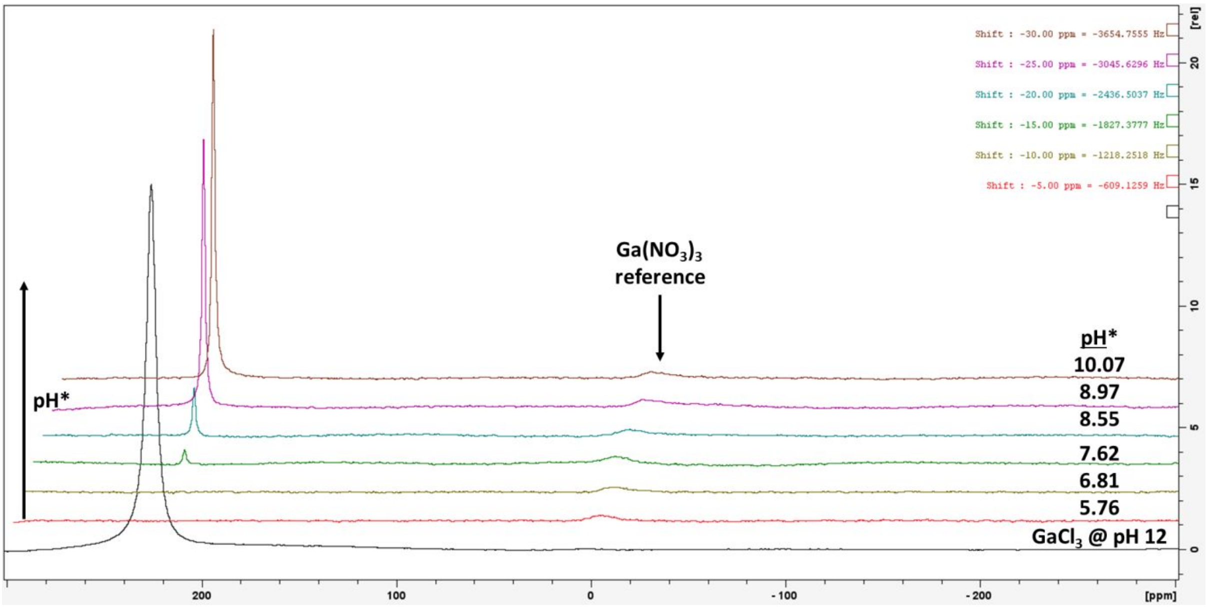

Figure S25: Offset ^{71}Ga NMR of [Ga(Tpaa)] in D_2O at various pH* values. $Ga(NO_3)_3$ in D_2O was added within a capillary as a reference. pH* values are indicated. $GaCl_3$ samples are included as references. (122 MHz, 298 K)

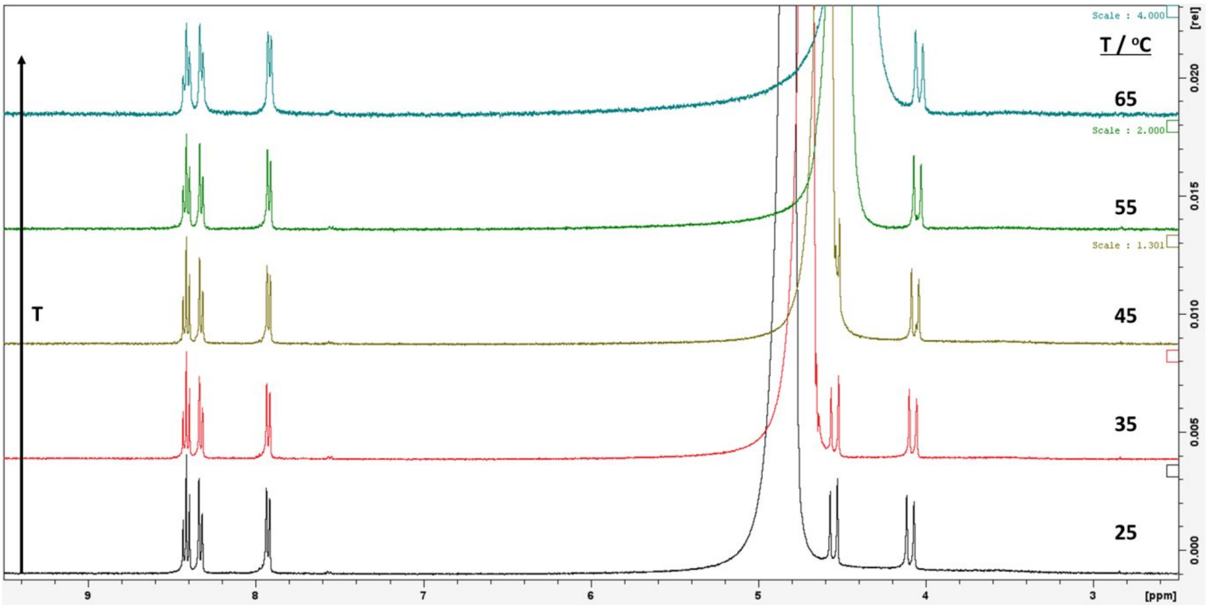

Figure S26: Variable temperature NMR of [Ga(Tpaa)]. Temperatures are indicated. (400 MHz, D_2O, pH* = 6.81)

9. References

[1] Táborský, P.; Lubal, P.; Havel, J.; Kotek, J.; Hermann, P.; Lukeš, I. Thermodynamic and Kinetic Studies of Lanthanide(III) Complexes with H_5do3ap (1,4,7,10-Tetraazacyclododecane-1,4,7-Triacetic-10-(Methylphosphonic Acid)), a Monophosphonate Analogue of H_4dota. *Collect. Czech. Chem. Commun*. **2005**, *70*, 1909–1942. DOI: 10.1135/cccc20051909.

[2] Försterová, M.; Svobodová, I.; Lubal, P.; Táborský, P.; Kotek, J.; Hermann, P.; Lukeš, I. Thermodynamic Study of Lanthanide(III) Complexes with Bifunctional Monophosphinic Acid Analogues of H_4dota and Comparative Kinetic Study of Yttrium(III) Complexes. *Dalton Transactions* **2007**, *0* (5), 535–549. DOI: 10.1039/B613404A.

[3] Notni, J.; Hermann, P.; Havlíčková, J; Kotek, J.; Kubíček, V.; Plutnar, J.; Loktionova, N.; Riss, P.J.; Rösch, F.; Lukeš, I., A Triazacyclononane-Based Bifunctional Phosphinate Ligand for the Preparation of Multimeric ^{68}Ga Tracers for Positron Emission Tomography, *Chem. Eur. J.* **2010**, *16*, 7174–7185. DOI: 10.1002/chem.200903281

[4] Kubíček, V.; Havlíčková, J.; Kotek, J.; Tircsó, G.; Hermann, P.; Tóth, É.; Lukeš, I., Gallium(III) Complexes of DOTA and DOTA−Monoamide: Kinetic and Thermodynamic Studies, *Inorg. Chem.* **2010**, *49*, 10960–10969. DOI: 10.1021/ic101378s

[5] Baes, C. F.; Mesmer, R. S. The Hydrolysis of Cations. In Berichte der Bunsengesellschaft & Physikalische Chemie; Berichte der Bunsengesellschaft & Physikalische Chemie; John Wiley & Sons: New York, London, Sydney, Toronto, **1977**; Vol. *81*, pp 245–246.

[6] A. E. Martell, R. M. Smith, *Critical Stability Constants*, Vols. 1–6, Plenum Press, New York, **1974–1989**

[7] NIST Standard Reference Database 46 (Critically Selected Stability Constants of Metal Complexes), Version 7.0, **2003**

[8] M. Kývala, I. Lukeš, *International Conference Chemometrics '95*, Pardubice, Czech Republic, **1995**, p. 63

[9] M. Kývala, P. Lubal, I. Lukeš, *IX. Spanish-Italian and Mediterranean Congress on Thermodynamics of Metal Complexes (SIMEC 98)*, Girona, Spain, **1998**. The full version of the OPIUM program is available (free of charge) on http://www.natur.cuni.cz/_kyvala/opium.html .

[10] Price, T.W.; Gallo, J.; Kubíček, V.; Böhmová, Z.; Prior, T.J.; Greenman, J.; Hermann, P.; Stasiuk, G.J., Amino acid based gallium-68 chelators capable of radiolabeling at neutral pH, *Dalton Trans.*, **2017**, 46, 16973-16982. DOI: 10.1039/C7DT03398B
